# Visual stream connectivity predicts assessments of image quality

**DOI:** 10.1167/jov.22.11.4

**Published:** 2022-10-11

**Authors:** Elijah F. W. Bowen, Antonio M. Rodriguez, Damian R. Sowinski, Richard Granger

**Affiliations:** 1 Brain Engineering Laboratory, Department of Psychological and Brain Sciences, Dartmouth, Hanover, NH, USA

**Keywords:** visual perception, similarity metrics, differential geometry, gestalt, scenes

## Abstract

Despite extensive study of early vision, new and unexpected mechanisms continue to be identified. We introduce a novel formal treatment of the psychophysics of image similarity, derived directly from straightforward connectivity patterns in early visual pathways. The resulting differential geometry formulation is shown to provide accurate and explanatory accounts of human perceptual similarity judgments. The direct formal predictions are then shown to be further improved via simple regression on human behavioral reports, which in turn are used to construct more elaborate hypothesized neural connectivity patterns. It is shown that the predictive approaches introduced here outperform a standard successful published measure of perceived image fidelity; moreover, the approach provides clear explanatory principles of these similarity findings.

## Introduction

**Figure 1.**
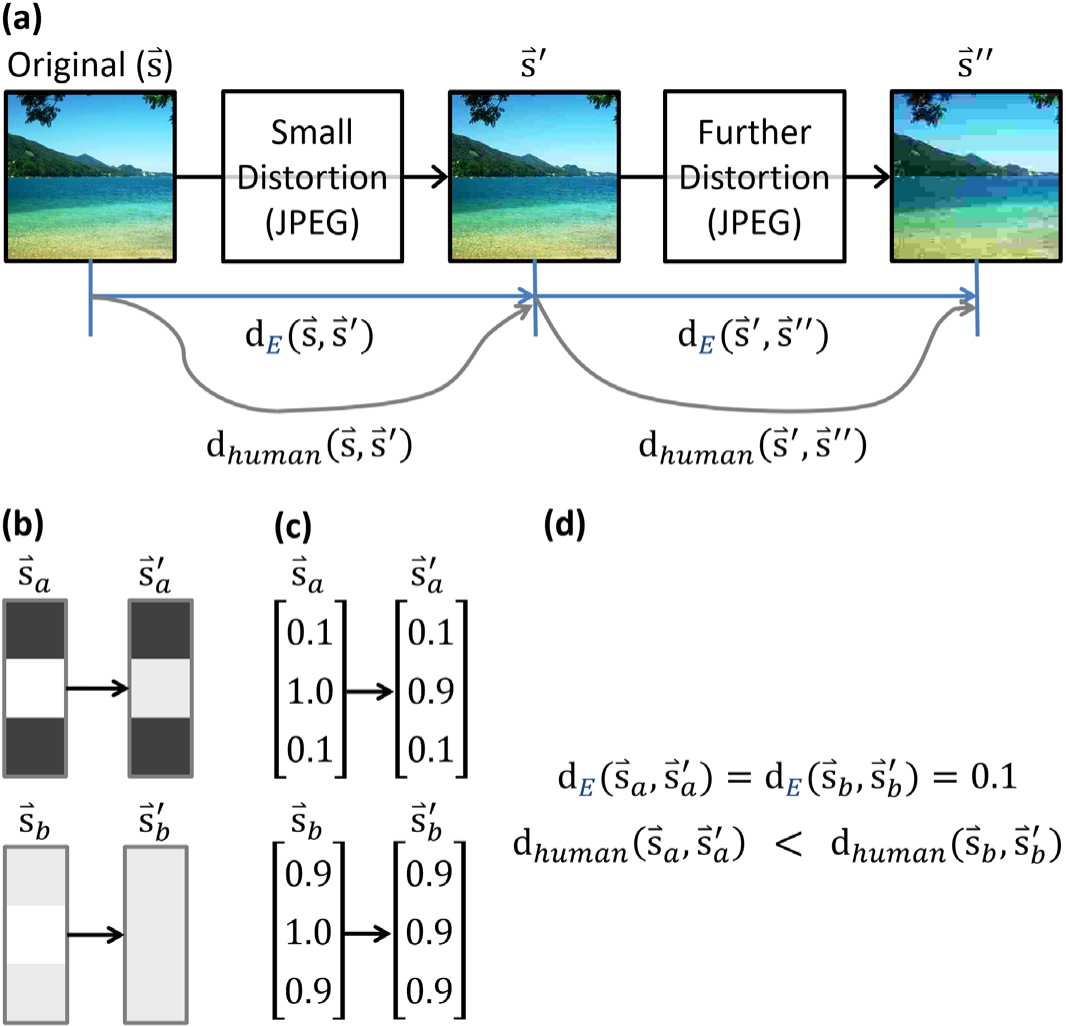
(a) As an original (non-degraded) image ($\overset{{}_{⇀}}{s}$) becomes increasingly compressed (via a lossy method such as JPEG), how dissimilar are the images judged to be? Equal physical changes, in terms of average luminance, will not be perceived as equal to humans. (b) A reduced example of three pixels in isolation (${\overset{{}_{⇀}}{s}}_{a}$) with degraded counterpart $\overset{{}_{⇀}}{s}{\;\;}_{a}^{'}$; for comparison, an alternate example (${\overset{{}_{⇀}}{s}}_{b}$) with degraded counterpart $\overset{{}_{⇀}}{s}{\;\;}_{b}^{'}$. (c) We can convert each image into a pixel vector of luminances (zero for black, one for white), as is often done. Euclidean distances can be computed between a pair of such image vectors by measuring luminance differences across each row and then combining the results. (d) The Euclidean distance between original and degraded images in both images is 0.1; however, humans overwhelmingly perceive change to be greater in the second (${\overset{{}_{⇀}}{s}}_{b}\to \overset{{}_{⇀}}{s}{\;\;}_{b}^{'}$) case, presumably due to the context of surrounding pixels.

To a human observer, two different images, warped by the same means (e.g., degraded by JPEG compression, ISO-10918) (Pennebaker & Mitchell, 1992; Wallace, 1992), may appear to have changed different amounts. In fact, prior work has shown that the perception of a warped image $\overset{{}_{⇀}}{s}$ does not cohere to any linear or univariate function of the mean change to pixel luminance (Dzhafarov & Colonius, 1999; Fechner, 1860; Fernandez & Farell, 2009; Georgiev, 2006; Itti & Koch, 2001; Oliva, Samengo, Leutgeb, & Mizumori, 2005; Petitot, 2003; Pons, Malo, Artigas, & Capilla, 1999; Sarti, Citti, & Petitot, 2008; Seung & Lee, 2000; Wang & Bovik, 2009). Figure 1 illustrates a sample discrepancy between the perceived change to an image and simple pixelwise vector distances. This neatly demonstrates the need for further accounting beyond a linear model. Elements of the image content interact within the visual system. The provocative question remains: What properties of percepts drive these interactions? A closer examination of such properties may help us to describe the underlying perceptual processes. Despite extensive study, the underlying perceptual mechanisms by which pixels give rise to similarity judgments remain unclear.

The field of full-reference image quality assessment (IQA) has endeavored to account for the perceived similarity of degraded images to their originals. Models of the psychophysics of just-noticeable differences and luminance masking describe perceived image degradation in terms of pixels (Daly, 1992; Damera-Venkata, Kite, Geisler, Evans, & Bovik, 2000; Egiazarian, Astola, Ponomarenko, Lukin, Battisti, & Carli, 2006; Larson & Chandler, 2010; Lukas & Budrikis, 1982; Ponomarenko, Silvestri, Egiazarian, Carli, Astola, & Lukin, 2007; Teo & Heeger, 1994; Wang & Bovik, 2002). Alternatively, models of saliency or attention have been used to weight perceptually important image regions (Farias & Akamine, 2012; Gu et al., 2016; Itti & Koch, 2001; Kuo, Su, & Tsai, 2016; Li & Bovik, 2010; Moorthy & Bovik, 2009; Moorthy & Bovik, 2011; Wang & Li, 2011; Wang & Shang, 2006; Xue, Zhang, Mou, & Bovik, 2014; Zhang, Shen, & Li, 2014). Among the most frequently cited works in this area, the Structural Similarity (SSIM) measure (Wang, Bovik, Sheikh, & Simoncelli, 2004; Wang, Simoncelli, & Bovik, 2003) combines metrics of pixel luminance, local contrast, and local correlation in normalized images. A commonality of these approaches is that each tests existing psychological principles using task-specific formulas. By contrast, this study seeks new, task-general pathways through which to link biology and psychophysics. We see degraded image perception as a question of perceptual geometry, which allows for the quantification of interactions between pixels. In other words, we seek to use perceptual geometry to compare the space of image stimuli (bitmaps) with the space of human image percepts in order to understand perception's mapping between the two.

Many tools from differential geometry can naturally capture the context-dependent processing of visual features (e.g., an individual pixel relative to its neighbors) (Figure 1). From the perspective of a simple and principle-agnostic parameterization—conditional relationships among pixels—one can quantify existing neural (Petitot, 2003) or psychophysical (Dzhafarov & Colonius, 1999; Georgiev, 2006; Sarti et al., 2008) principles. Such quantifications in the framework of perceptual geometry are indeed predictive of behavior. Consider, for example, Pons and colleagues (1999), who quantified local contrast geometrically and successfully used the resultant model in IQA. The formalism that Pons and colleagues and Malo, Ferri, Albert, Soret, and Artigas (2000) pioneered in IQA has been extended by others. Laparra, Muñoz-Marí, and Malo (2010) introduced modeling mechanisms for the use of divisive normalization in perceptual geometry. Others have fused perceptual geometry with the geometry of natural image statistics, modeling both jointly (Epifanio, Gutierrez, & Malo, 2003; Malo, Epifanio, Navarro, & Simoncelli, 2005). Others have proposed metrics derived from natural image statistics instead of biology or psychophysics (Berardino, Laparra, Ballé, & Simoncelli, 2017; da Fonseca & Samengo, 2016). Although the objective of these metrics diverges slightly, the underlying mathematics is related (da Fonseca & Samengo, 2018). Perceptual spaces are often surprisingly accessible to inference. One can use the same geometric tools to compare multiple hypotheses (e.g., Georgiev, 2006; Petitot, 2003), as in this paper; generate new hypotheses (e.g., Dzhafarov & Colonius, 1999); and even integrate disparate hypotheses into a single underlying principle (Rodriguez & Granger, 2021).

We construct a first-principles framework based upon similarity spaces and data-driven modeling. A similarity space quantifies an item based on its similarity to other items, eschewing positional coordinate systems. This framework has proven crucial to illuminating underlying processes in human perception. For example, neural codes have been shown to form similarity spaces, in which the similarity among population activity patterns is more interpretable than any individual pattern (de Beeck, Wagemans, & Vogels, 2001; Haushofer, Livingstone, & Kanwisher, 2008; Kriegeskorte & Kievit, 2013; Oliva et al., 2005). Similarity spaces have proved a valuable way to quantify hypotheses of visual object and shape perception (Ashby & Perrin, 1988; Edelman, 1998; Edelman & Shahbazi, 2012; Ehm & Wackermann, 2012; Goldstone, 1994; Unzicker, Jüttner, & Rentschler, 1998). In fact, it has been posited that similarity spaces may even be a primary product of perception (Edelman, 1998; Shahbazi, Raizada, & Edelman, 2016).

Presumably, human judgments of visual similarity among images approximate samples from a perceiver's internal similarity space (de Beeck et al., 2001; Medin, Goldstone, & Gentner, 1993). Treating human behavioral judgments as such, we construct a model of the *strain* (as used in physics) involved in converting Euclidean image similarity into perceptual image similarity. We then derive an image-space similarity measure that matches. We show that straightforward properties of circuitry in the early visual pathway directly give rise to derived non-Euclidean similarity measures. These similarity measures are predictive of human behavioral responses, providing a link between early visual circuitry and behavior. The results are compatible with findings in the literatures of psychophysics (e.g., Oliva et al., 2005; Yue, Biederman, Mangini, von der Malsburg, & Amir, 2012) and IQA (e.g., Pons et al., 1999). Moreover, the formulation has already been shown to account for a seemingly unrelated set of visual psychophysics phenomena (i.e., crowding) (Rodriguez & Granger, 2021). We believe that this formalism is the first to use strain to directly approach the possible causal relationships between biological connectivity and psychophysics. In summary, this formalism may be used to refine our understanding of the unseen processes that give way to the quirks of human visual perception and ultimately prove useful to future applications in predicting judgments and behavior.

## Theoretical treatment

### A differential geometry of image perception

Bitmaps, like photoreceptors in the eye, simplistically encode images onto Cartesian coordinates of pixels. Each axis can denote one (independent) pixel in an image, and the value along an axis is the luminance of that pixel. To calculate the dissimilarity between two image stimuli, $\overset{{}_{⇀}}{s}$ and $\overset{{}_{⇀}}{s}\;\;{}^{'}$, a straw-man approach is to simply assume that the change in each bitmap pixel is independently processed and then averaged (as in mean squared error [MSE]):

(1) $$\begin{array}{c} \frac{1}{D}\sum_{d=1}^{D}\left(s^{'}\;{\;}_{d}-s_{d}\right)\left(s^{'}\;{\;}_{d}-s_{d}\right)\; \end{array}$$

where *D* is the number of pixels in the image. This can be rewritten, in linear algebra terms, as the dot product of the vector between $\overset{{}_{⇀}}{s}$ and $\overset{{}_{⇀}}{s}\;\;{}^{'}$. Dropping the 1/*D* term (which is constant in each dataset) yields a measure of image difference which is the (squared) Euclidean distance:

(2) $$\begin{array}{c} d_{E}^{2}\left(\overset{{}_{⇀}}{s},\overset{{}_{⇀}}{s}\;\;{}^{'}\right)={\left(\overset{{}_{⇀}}{s}\;\;{}^{'}-\overset{{}_{⇀}}{s}\right)}^{T}\left(\overset{{}_{⇀}}{s}\;\;{}^{'}-\overset{{}_{⇀}}{s}\right)\; \end{array}$$

Perceptual similarity is best described by image-space metrics that are non-Euclidean (Oliva et al., 2005; Petitot, 2003; Resnikoff, 1974; Sarti et al., 2008). In psychophysics, the relationship between Euclidean and perceived distance is often reliable (if complicated). This affords an opportunity to model similarity judgments as a structured deviation from Euclidean distance (Oliva et al., 2005; Petitot, 2003; Pons et al., 1999; Resnikoff, 1974; Sarti et al., 2008; Seung & Lee, 2000). We present an analysis in which this neural transformation is modeled under continuum mechanics (Landau & Lifshitz, 1986)—in this framework, a displacement of images $\overset{{}_{⇀}}{s}$ to new “perceived” positions ${\overset{{}_{⇀}}{s}}_{P}$ within an image space. Each new position differs from the original via a *displacement field*, $\overset{{}_{⇀}}{u}\left(\overset{{}_{⇀}}{s}\right)$:

(3) $$\begin{array}{c} {\overset{{}_{⇀}}{s}}_{P}=\overset{{}_{⇀}}{s}+\overset{{}_{⇀}}{u}\left(\overset{{}_{⇀}}{s}\right)\; \end{array}$$

**Figure 2.**
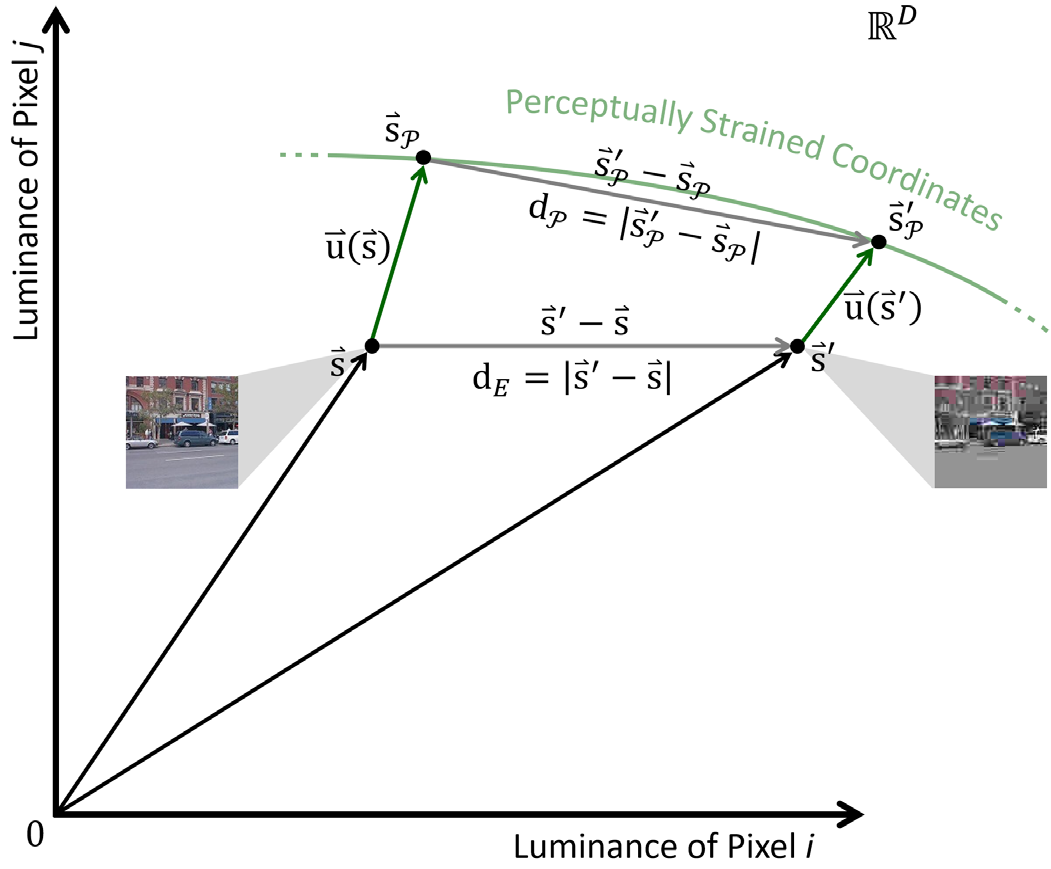
Vector space account of perceptual strain. Each possible image can be considered a point (i.e., a vector from the origin; black arrows) in pixelated image space, where each Cartesian coordinate is the luminance of one pixel. Here, we plot only two such coordinate axes for simplicity. When humans perceive images, cells form population codes that change the representations of the light patterns. Therefore, an image $\overset{{}_{⇀}}{s}$ and its degraded counterpart $\overset{{}_{⇀}}{s}{}^{'}$ are displaced to new coordinates ${\overset{{}_{⇀}}{s}}_{P}$ and $\overset{{}_{⇀}}{s}{}^{'}\;{\;}_{P}$. This perceptual strain is quantified as a vector field $\overset{{}_{⇀}}{u}\left(\overset{{}_{⇀}}{s}\right)$ that can be evaluated at any image (green arrows). Approach I defines $\overset{{}_{⇀}}{u}\left(\overset{{}_{⇀}}{s}\right)$ in terms of biological connectivity patterns. Approach II triangulates the vector field of perceptual strain from Euclidean $\left(d_{E}\right)$ and perceived ($d_{P}$) distance measurements. Crucially, in our hands, perceived distance is Euclidean after the correct perceptual displacement field is applied to images. The new image positions (${\overset{{}_{⇀}}{s}}_{P}$) are left as an internal property of neural representations.

Perception strains the image space, which changes the Euclidean distances between stimuli. Figure 2 lays out the problem in pixelated image space. The perceived difference between $\overset{{}_{⇀}}{s}$ and $\overset{{}_{⇀}}{s}\;\;{}^{'}$ (the Euclidean distance between ${\overset{{}_{⇀}}{s}}_{P}$ and $\overset{{}_{⇀}}{s}\;\;{}^{'}\;{\;}_{P}$) is

(4) $$\begin{array}{c} d_{P}^{2}\left(\overset{{}_{⇀}}{s},\overset{{}_{⇀}}{s}{}^{'}\right)={\left(\overset{{}_{⇀}}{s}{}^{'}\;{\;}_{P}-{\overset{{}_{⇀}}{s}}_{P}\right)}^{T}\;\left(\overset{{}_{⇀}}{s}{}^{'}\;{\;}_{P}-{\overset{{}_{⇀}}{s}}_{P}\right)\; \end{array}$$

We seek to formalize the geometry of perceptual space—how points compare to one another. Geometry is agnostic to the exact value of this new position, ${\overset{{}_{⇀}}{s}}_{P}$. Therefore, we will focus on constructing a new measure of image difference that is a function of the original images (not of ${\overset{{}_{⇀}}{s}}_{P}$). We now show that the displacement in Equation 3 can be reinterpreted as distortions of the metric of the space in which the images live. Each difference vector between a perceived image and a perceived degraded copy is defined as

(5) $$\begin{array}{c} \overset{{}_{⇀}}{s}\;\;{}^{'}\;{\;}_{P}-{\overset{{}_{⇀}}{s}}_{P}=\overset{{}_{⇀}}{s}\;\;{}^{'}+\overset{{}_{⇀}}{u}\left(\overset{{}_{⇀}}{s}\;\;{}^{'}\right)-\overset{{}_{⇀}}{s}-\overset{{}_{⇀}}{u}\left(\overset{{}_{⇀}}{s}\right)\; \end{array}$$

Taylor expanding to first order, Equation 5 reads as a function of how the displacement field has changed between $\overset{{}_{⇀}}{s}$ and $\overset{{}_{⇀}}{s}{}^{'}$ (how strain changes as images change, ${\overset{{}_{⇀}}{\nabla }}_{s}\overset{{}_{⇀}}{u}$):

(6) $$\begin{array}{c} \overset{{}_{⇀}}{s}\;\;{}^{'}\;{\;}_{P}-{\overset{{}_{⇀}}{s}}_{P}=\left(\overset{{}_{⇀}}{s}\;\;{}^{'}-\overset{{}_{⇀}}{s}\right)+{\left({\overset{{}_{⇀}}{\nabla }}_{s}{\overset{{}_{⇀}}{u}}^{T}\right)}^{T}\left(\overset{{}_{⇀}}{s}\;\;{}^{'}-\overset{{}_{⇀}}{s}\right)\; \end{array}$$

A first-order Taylor polynomial models the displacement field between $\overset{{}_{⇀}}{s}$ and $\overset{{}_{⇀}}{s}\;\;{}^{'}$ as a linear function. This first-order approximation does not account for how the gradient of strain *changes* along the path from $\overset{{}_{⇀}}{s}$ to $\overset{{}_{⇀}}{s}\;\;{}^{'}$. The approximation will be poor if the path from $\overset{{}_{⇀}}{s}$ to $\overset{{}_{⇀}}{s}\;\;{}^{'}$ is sufficiently nonlinear. Two conditions can guarantee an accurate approximation. First, the displacement field can have little curvature relative to the distance between images. Second, the distance between images can be sufficiently small to make any curvature irrelevant. Although the degradations that we evaluate (see Methods) are at times obvious to subjects, they are miniscule on the scale of image space. That is, degradations never transform one reference image into another one, or make nonlocal changes. In the IQA task, we believe that both conditions can be taken as reasonable assumptions, at the cost of some modeling error. Per the results, even an imprecise first-order approximation appears to capture valuable patterns. An important next step will be to utilize highly nonlinear models of perceptual strain, building on important prior work (Epifanio et al., 2003; Laparra et al., 2010; Malo et al., 2000; Malo et al., 2005; Pons et al., 1999).

Equation 6 can be factored as

(7) $$\begin{array}{c} \overset{{}_{⇀}}{s}{}^{'}\;{\;}_{P}-{\overset{{}_{⇀}}{s}}_{P}=\left(I+{\left({\overset{{}_{⇀}}{\nabla }}_{s}{\overset{{}_{⇀}}{u}}^{T}\right)}^{T}\right)\left(\overset{{}_{⇀}}{s}{}^{'}-\overset{{}_{⇀}}{s}\right)\; \end{array}$$

where **I** is the identity matrix (the tensor of Cartesian coordinates in Euclidean space); ${\overset{{}_{⇀}}{\nabla }}_{s}{\overset{{}_{⇀}}{u}}^{T}$ is a matrix where the value in the *i*th row and *j*th column and ∂*u_i_*/∂*s_j_*, describe how much additional displacement the luminance change to pixel *j* contributes to the perceptual displacement of pixel *i*:

(8) $$\begin{array}{c} {\left({\overset{{}_{⇀}}{\nabla }}_{s}{\overset{{}_{⇀}}{u}}^{T}\right)}^{T}\equiv \left[\begin{array}{ccc} \frac{\partial u_{1}}{\partial s_{1}} & \cdots & \frac{\partial u_{1}}{\partial s_{D}}\\ \vdots & \ddots & \vdots \\ \frac{\partial u_{D}}{\partial s_{1}} & \cdots & \frac{\partial u_{D}}{\partial s_{D}} \end{array}\right]\; \end{array}$$

A high value of ∂*u_i_*/∂*s_j_* suggests a strong connection between pixels *i* and *j*. The Euclidean distance metric in Cartesian coordinates of pixels (e.g., Equations 2 and 4) has the identity matrix as its tensor. Now that we understand Equation 7, we can compute perceived distance (Equation 4) without reference to ${\overset{{}_{⇀}}{s}}_{P}$:

(9) $$\begin{array}{ccc} d_{P}^{2}\left(\overset{{}_{⇀}}{s},\overset{{}_{⇀}}{s}{}^{'}\right) & = & {\left(\overset{{}_{⇀}}{s}{}^{'}-\overset{{}_{⇀}}{s}\right)}^{T}{\left(\;I+{\left({\overset{{}_{⇀}}{\nabla }}_{s}{\overset{{}_{⇀}}{u}}^{T}\right)}^{T}\;\right)}^{T}\;I\\ & & \times \left(\;I+{\left({\overset{{}_{⇀}}{\nabla }}_{s}{\overset{{}_{⇀}}{u}}^{T}\right)}^{T}\;\right)\left(\overset{{}_{⇀}}{s}{}^{'}-\overset{{}_{⇀}}{s}\right)\; \end{array}$$

See Derivation of perceptual distance for a derivation. Equation 9 can be found in other works of perceptual geometry, each of which defines the non-Euclidean metric in terms of what an expert will recognize as a Jacobian (defined in the following sections) that models stimulus response. Here, the Jacobian represents how strain changes across images. See, for example, Pons and colleagues (1999, equations 4 and 16); Malo and colleagues (2000, equation 1); Epifanio and colleagues (2003, equations 2 and 9); Malo and colleagues (2005, equations 5 and 6); and Laparra and colleagues (2005, equations 8 and 10).

The transition from Equation 4 to Equation 9 is a crucial step, because it alleviates the need for ${\overset{{}_{⇀}}{s}}_{P}$, which ${\overset{{}_{⇀}}{s}}_{P}$ is the observer's internal percept and the object of study but not directly measurable. So far, the perceptual displacement field has been equally immeasurable. In the next section, we will discuss how the perceptual displacement field (our central quantification of perceptual strain) can be related to biological connectivity and psychophysical measurements.

### Mathematical relationship between biological projection patterns and perceptual strain

We define perception **P** as an operation that changes each bitmap image stimulus $\overset{{}_{⇀}}{s}$ (a point on Cartesian coordinates of pixels) to a perceived vector ${\overset{{}_{⇀}}{s}}_{P}$:

(10) $$\begin{array}{c} {\overset{{}_{⇀}}{s}}_{P}=P\overset{{}_{⇀}}{s}\; \end{array}$$

**Figure 3.**
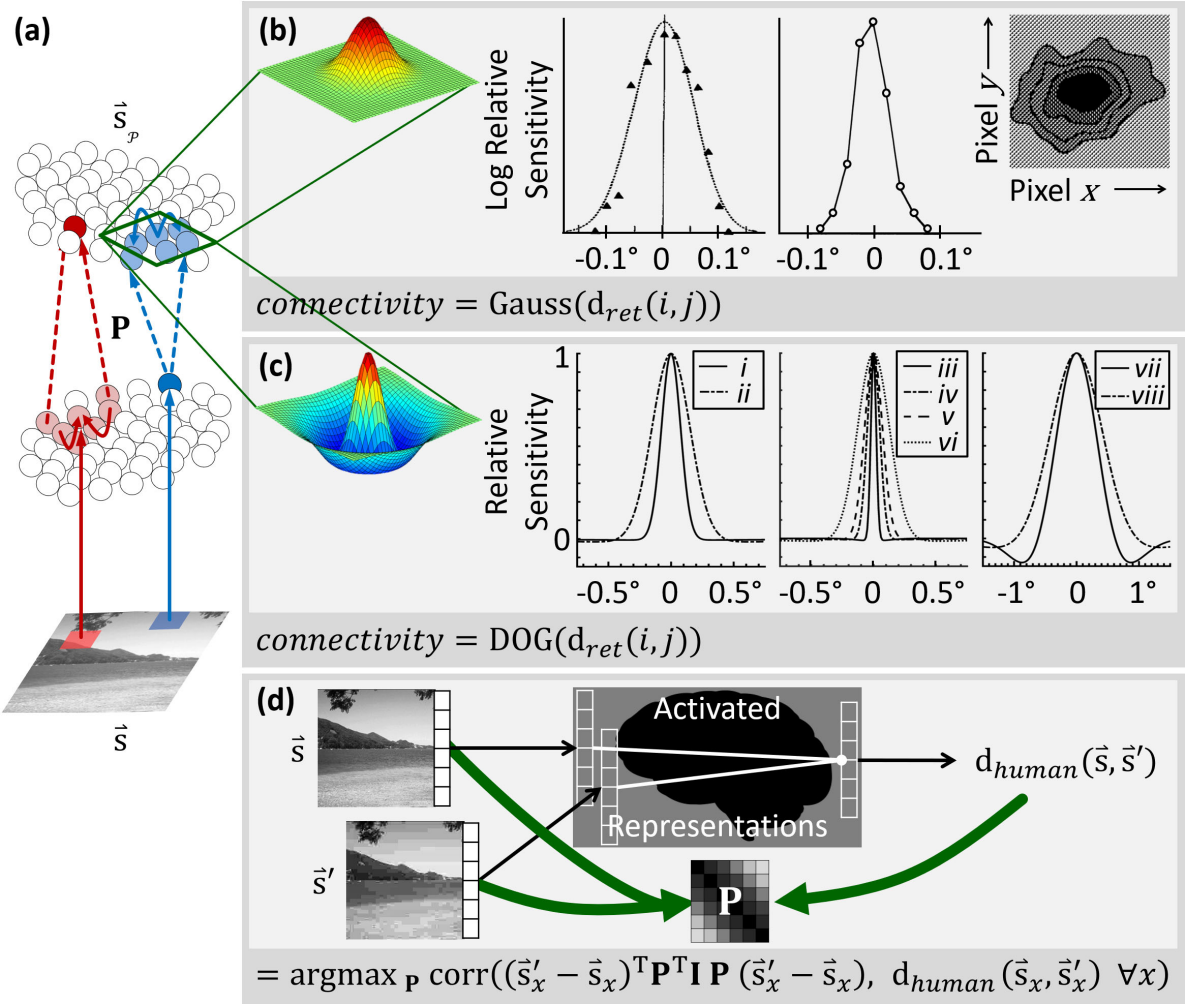
Principles formalized from neural connectivity. (a) Caricature of neural projections from cells in the lower population (where each cell represents light at one location, much like pixels) to downstream cells, with a topographic connectivity pattern. Neighboring cells represent neighboring pixels. These cells connect with their neighbors (solid arrows). Downstream, cells again receive projections from neighbors (dashed arrows). See text. (b) A Gaussian connectivity function (see text). Such connectivity is often found in (left to right) retinal ganglion cells (De Monasterio, 1978; Young, 1987) and visual cortex (Young & Lesperance, 2001). (c) A difference-of-Gaussian connectivity function (see text). Such connectivity is found in, for example, OFF-center retinal bipolar cells (*i*, midget; *ii*, diffuse (Dacey et al., 2000)). Similar connectivity is also found in retinal ganglion cells that project to the parvocellular (*iii*, 0°–5°; *iv*, 10°–20° from visual center) (Croner & Kaplan, 1995) or magnocellular (*v*, 0°–10°; *vi*, 10°–20° from visual center) (Croner & Kaplan, 1995) pathways, across the visual field: *vii*, peripheral (Dacey, 1996; Dacey, 2000) and *viii* < 10° from fixation (Rodieck, 1965). (d) Instead of drawing connectivity patterns from documented biology, we can use tools such as regression to find the pattern of connectivity that, given simplicity assumptions, best explains the relationship between images and human ratings.

Let us say that **P** is a locally multilinear operator—a *D* × *D* matrix for each stimulus. The main diagonal represents 1:1 topographic connectivity among neurons, or an unmodified percept. Each off-diagonal describes an additional biological connection or perceptual interaction (that may strain image space). This matrix is a simple way to quantify connectomes and local projection patterns like those in Figure 3. Here, each element of **P** is a scalar function describing how a pair of neurons, receptive fields, concepts, or brain regions relate. This concept of connectivity is believed to be equivalent to some quantifications of linear cell receptive fields (Chichilnisky, 2001). Qualitatively, the incoming connectivity to a neuron defines its receptive field.

In the retina, image pixels are topographically mapped to photoreceptors—adjacent pixels are processed by adjacent photoreceptors. In turn, neighboring photoreceptors project to neighboring retinal cells, with some lateral excitation and inhibition (Figure 3a). Thus, a given retinal cell receives information from a small contiguous region of an image (Dacey, Packer, Diller, Brainard, Peterson, & Lee, 2000). The resulting receptive fields are typically fit by a Gaussian function of relative retinotopic position between points *i* and *j* on an image (retinal distance, *d_ret_*(*i*, *j*) (Dacey et al., 2000; De Monasterio, 1978; Sincich & Blasdel, 2001; Young, 1987; Young & Lesperance, 2001); for electrophysiological examples, see Figure 3b:

(11) $$\begin{array}{c} \mathrm{Gauss}\left(d_{ret}\left(i,j\right)\right)=\exp\left(\frac{-d_{ret}{\left(i,j\right)}^{2}}{2\sigma^{2}}\right)\; \end{array}$$

The early visual stream conserves this topographic connectivity (Croner & Kaplan, 1995; Dacey, 1996; Dacey, 2000; Dacey & Petersen, 1992; Dacey et al., 2000; Hubel & Wiesel, 1962; Martinez & Alonso, 2003; Olshausen, 2003; Rodieck, 1965; Von der Malsburg, 1973). This preserved topography may be expressed using Gaussian and center-surround connectivity. The latter can be approximated as the difference between two concentric Gaussian functions of distance—a narrow center and broad surround (difference of Gaussians [DOG]) (Figure 3c):

(12) $$\begin{array}{ccc} \;\mathrm{DOG}\left(d_{ret}\left(i,j\right)\right) & \;= & \frac{1}{1+\alpha }\exp\left(\frac{-d_{ret}{\left(i,j\right)}^{2}}{2\sigma_{\mathrm{center}}^{2}}\right)\\ \; & & -\frac{\alpha }{1+\alpha }\exp\left(\frac{-d_{ret}{\left(i,j\right)}^{2}}{2\sigma_{\mathrm{surround}}^{2}}\right)\; \end{array}$$

Equations 11 and 12 describe how pixels will be combined when these cells process an image. Equation 12 is the last biological function that we require.

By connecting neuroscience to psychophysics, we can begin to generate new predictions and understandings of how the underlying biology explains behavior. We are ready to specify how perceptual strain links these two bodies of knowledge. We set the biological projection in Equation 10 equal to continuum mechanics Equation 3 (and rearrange), yielding:

(13) $$\begin{array}{c} \overset{{}_{⇀}}{u}\left(\overset{{}_{⇀}}{s}\right)=P\overset{{}_{⇀}}{s}\;-\overset{{}_{⇀}}{s}\; \end{array}$$

However, to measure distance, we wish to relate **P** to the *gradient* of $\overset{{}_{⇀}}{u}$:

(14) $$\begin{array}{c} {\overset{{}_{⇀}}{\nabla }}_{s}{\overset{{}_{⇀}}{u}}^{T}={\overset{{}_{⇀}}{\nabla }}_{s}\left({\overset{{}_{⇀}}{s}}^{T}P^{T}-{\overset{{}_{⇀}}{s}}^{T}\right)\; \end{array}$$

The gradient of $\overset{{}_{⇀}}{s}$ with respect to itself is simply **I**. We make this replacement and apply a transpose, returning the left side to something more simply expressed:

(15) $$\begin{array}{c} {\left({\overset{{}_{⇀}}{\nabla }}_{s}{\overset{{}_{⇀}}{u}}^{T}\right)}^{T}=P-I\; \end{array}$$

Equation 15 shows that any known perceptual operator **P** can be written in terms of the derivative of the displacement field. We can say that the displacement field is *generated by* the perceptual operator.

Psychophysicists often measure scalar differences between end percepts. To relate these difference measures to biology, we need a formula for difference as a function of **P**. Using Equation 15, we can insert **P** into Equation 9. The perceived difference between $\overset{{}_{⇀}}{s}$ and $\overset{{}_{⇀}}{s}{}^{'}$ is simply

(16) $$\begin{array}{c} d_{P}^{2}\left(\overset{{}_{⇀}}{s},\overset{{}_{⇀}}{s}{}^{'}\right)={\left(\overset{{}_{⇀}}{s}{}^{'}-\overset{{}_{⇀}}{s}\right)}^{T}P^{T}I\;P\;\left(\overset{{}_{⇀}}{s}{}^{'}-\overset{{}_{⇀}}{s}\right)\; \end{array}$$

where image difference has been distorted by perceptual strain. In terms of differential geometry, **P** is the Jacobian of the perceptual distortion. If there exists no perceptual strain, **P** = **I**, and $d_{P}^{2}\left(\overset{{}_{⇀}}{s},\overset{{}_{⇀}}{s}{}^{'}\right)=d_{E}^{2}\left(\overset{{}_{⇀}}{s},\overset{{}_{⇀}}{s}{}^{'}\right)$.

Given **P**, Equation 16 can be used to calculate the perceived difference without direct measurement of the displacement field. Instead, Equation 16 predicts perceived difference using a perceptual strain that has been inferred from biological projection patterns. In the next section, we will introduce two approaches for selecting **P**.

### Predicting perceived distance

Equation 15 is a simple way to generate the perceptual displacement field from **P**. We evaluate several possible forms of **P** herein. The first is Gaussian connectivity between the cells that favor pixels *i* and *j* (as described earlier and in Figure 3b):

(17) $$\begin{array}{c} p_{i,j\mathrm{Gauss}}=\mathrm{Gauss}\left(d_{ret}\left(i,j\right)\right)\; \end{array}$$

For a perceptual operator using difference of Gaussians, a simple change to Equation 17 suffices:

(18) $$\begin{array}{c} p_{i,j\mathrm{DOG}}=\mathrm{DOG}\left(d_{ret}\left(i,j\right)\right)\; \end{array}$$

For convenience, we designed the Gaussian form in Equation 11 to provide Gauss(0) = 1. In fact, when choosing our units, we set all perceptual relations relative to the center of the receptive field. Each **P** has 1’s along the diagonal. When we subtract **I** in Equation 15, we zero the diagonal elements of ${\left({\overset{{}_{⇀}}{\nabla }}_{s}{\overset{{}_{⇀}}{u}}^{T}\right)}^{T}$ (and thus the strain tensor; see Derivation of strain tensor). Together, these components account for image space dilation, which cannot be measured using relative psychophysical distances. (Diagonal connectivity, the Euclidean component of perception, is separately represented by **I** in Equation 15.)

It will be seen in the Results and predictive capacity section that both Gaussian and DOG versions of this equation, with no further modifications, provide unexpectedly accurate predictions of human image similarity judgments. In fact, these very surprisingly outperform an approach that is designed specifically for the task (Figure 7, Table 1).

**Table 1.** Pearson correlation with humans. Pearson correlation with humans (DMOS) on log–log axes for the presented models (rows) on several datasets (columns). Euclidean distance (MSE) between images and models from the literature (e.g., SSIM) are included for comparison. Except for SceneIQ, correlations were calculated on entire datasets. SceneIQ Online and SceneIQ Lab correlations represent the mean across two random folds of original (non-degraded) images (see Methods). Approach II SceneIQ Online was trained on each fold of SceneIQ Online and tested on the other fold. On other datasets, the reported correlation represents approach II fitted to all SceneIQ Online and tests the generalization of approach II trained on SceneIQ Online to a different dataset. For each dataset, the highest-performing model is indicated in bold and the second highest with an underline. All correlations were found to differ from zero with *p* << 0.001 via permutation test (after Bonferroni correction for 100 comparisons). C = coast, F = forest, H = highway, IC = inside city, M = mountain, OC = open country, S = street, TB = tall building, MS-SSIM = multi-scale structural similarity, IW-SSIM = image content weighted structural similarity, VSNR = visual signal-to-noise ratio, VIF = visual information fidelity, VIFP = pixel-based visual information fidelity, IFC = information fidelity criterion, GMSD = gradient magnitude similarity deviation.

	CSIQ		TID2013			SceneIQ Online									
	JPEG	Revised	JPEG	Mean	Toyama JPEG	All	C	F	H	IC	M	OC	S	TB	SceneIQ Lab all
Correlation on log–log axes															
Approach I															
Gaussian σ = 0.6 px (0.0310°)	0.93	0.76	0.79	0.71	0.42	0.63	0.63	0.75	0.61	0.79	0.67	0.63	0.79	0.67	0.67
Gaussian σ = 2 px (0.1238°)	0.95	0.92	0.49	0.53	0.32	0.56	0.54	0.68	0.52	0.69	0.55	0.51	0.68	0.50	0.55
Center surround (DOG)	0.95	0.92	0.96	0.81	0.52	0.83	0.85	0.85	0.85	0.86	0.84	0.85	0.85	0.83	0.87
Approach II															
SceneIQ Online	0.94	0.91	0.95	0.82	0.76	0.84	**0.86**	0.86	0.86	0.87	0.86	**0.86**	0.87	0.83	0.89
SceneIQ Lab	0.94	0.90	0.95	**0.82**	0.76	—	—	—	—	—	—	—	—	—	0.88
Euclidean (MSE)	0.87	0.55	0.86	0.78	0.41	0.45	0.54	0.51	0.57	0.71	0.54	0.49	0.73	0.63	0.52
SSIM	0.86	0.78	0.88	0.72	0.61	0.65	0.68	0.81	0.64	0.80	0.75	0.75	0.82	0.75	0.70
MS-SSIM	0.86	0.88	0.89	0.70	0.78	0.77	0.78	0.80	0.81	0.82	0.82	0.81	0.83	0.79	0.80
IWSSIM	0.86	0.89	0.86	0.68	0.82	0.75	0.75	0.78	0.81	0.81	0.82	0.79	0.83	0.77	0.77
VSNR	0.88	0.76	0.92	0.71	0.80	0.68	0.65	0.71	0.75	0.80	0.69	0.62	0.76	0.75	0.73
VIF	**0.96**	**0.96**	0.94	0.77	**0.88**	0.85	0.85	**0.87**	0.87	**0.88**	**0.88**	0.86	**0.88**	0.85	0.89
VIFP	0.95	0.89	0.92	0.76	0.75	0.78	0.77	0.83	0.84	0.84	0.84	0.81	0.86	0.81	0.83
IFC	0.87	0.79	0.82	0.60	0.82	0.71	0.75	0.81	0.78	0.80	0.78	0.77	0.84	0.74	0.72
GMSD	0.94	0.94	**0.96**	0.82	0.79	**0.86**	0.86	0.86	**0.87**	0.87	0.88	0.86	0.86	**0.85**	**0.89**
PerceptNet	0.88	0.71	0.66	0.64	0.33	0.41	0.44	0.48	0.49	0.55	0.35	0.35	0.64	0.42	0.46
BioMultilayer	0.93	0.85	0.93	0.77	0.73	0.76	0.75	0.81	0.79	0.81	0.77	0.81	0.84	0.77	0.79
Correlation on linear axes															
Approach I															
Gaussian σ = 0.6 px (0.0310°)	0.93	0.76	0.80	0.72	0.42	0.63	0.63	0.75	0.61	0.79	0.67	0.64	0.80	0.67	0.66
Gaussian σ = 2 px (0.1238°)	0.94	0.92	0.45	0.51	0.31	0.50	0.50	0.65	0.46	0.66	0.49	0.46	0.65	0.45	0.48
Center surround (DOG)	0.93	0.91	0.96	0.82	0.50	0.83	0.84	0.86	0.85	0.86	0.84	0.85	0.85	0.83	0.87
Approach II															
SceneIQ Online	0.76	0.81	0.90	0.74	0.70	0.76	0.75	0.81	0.75	0.81	0.78	0.80	0.80	0.77	0.79
SceneIQ Lab	0.76	0.80	0.90	0.75	0.70	—	—	—	—	—	—	—	—	—	0.79
Euclidean (MSE)	0.86	0.55	0.86	0.78	0.40	0.45	0.54	0.51	0.58	0.71	0.54	0.50	0.74	0.63	0.51
SSIM	0.84	0.78	0.87	0.70	0.60	0.63	0.67	0.80	0.64	0.80	0.75	0.74	0.82	0.75	0.69
MS-SSIM	0.87	0.89	0.91	0.71	0.79	0.79	0.79	0.82	0.83	0.83	0.83	0.82	0.84	0.80	0.81
IWSSIM	0.87	0.90	0.87	0.69	0.84	0.76	0.75	0.79	0.82	0.82	0.83	0.80	0.84	0.78	0.77
VSNR	0.88	0.76	0.92	0.72	0.80	0.68	0.66	0.72	0.76	0.80	0.69	0.62	0.76	0.76	0.73
VIF	0.90	0.94	0.88	0.71	**0.88**	0.80	0.78	0.84	0.84	0.86	0.85	0.82	0.87	0.83	0.83
VIFP	0.91	0.88	0.88	0.72	0.74	0.75	0.72	0.80	0.82	0.84	0.82	0.79	0.85	0.80	0.79
IFC	0.79	0.73	0.76	0.57	0.82	0.65	0.69	0.78	0.76	0.79	0.75	0.72	0.83	0.73	0.67
GMSD	**0.94**	**0.94**	**0.97**	**0.83**	0.81	**0.87**	**0.87**	**0.87**	**0.88**	**0.88**	**0.88**	**0.87**	**0.87**	**0.86**	**0.90**
PerceptNet	0.80	0.66	0.60	0.62	0.28	0.36	0.39	0.51	0.41	0.48	0.31	0.31	0.61	0.37	0.39
BioMultilayer	0.93	0.85	0.94	0.78	0.74	0.77	0.76	0.82	0.80	0.82	0.78	0.82	0.85	0.78	0.79

**P**_Gauss_ (Equation 17) and **P**_DOG_ (Equation 18) are used as examples of “approach I” herein. This simple approach produces a Jacobian from the displacement field, which lets us measure the perceived distance between two stimuli. The resulting Jacobians are of course unlikely to be perfectly accurate representations of the actual connectivity patterns in early visual pathways, which are shaped by development and learning.

Thus, in a second approach (“approach II”), we regress on pairs of image change ($\overset{{}_{⇀}}{s}{}^{'}-\overset{{}_{⇀}}{s}$) and human evaluations of dissimilarity. We vary each cell of the perceptual Jacobian until the resulting tensor produces image dissimilarities that are locally maximally Pearson-correlated with human ratings. The resulting Jacobian may be hypothesized to more accurately correspond to the transforms that may be taking place along the connections in the early visual pathway, as in Figure 3d. This approach may be considered roughly accordant with methods in the IQA literature that attempt to learn optimal predictors of human judgments (e.g., Narwaria & Lin, 2010; Shnayderman, Gusev, & Eskicioglu, 2006). Using approach II, we can model the pattern of connectivity that, given simplicity assumptions, best explains how images relate to human ratings. Our objective is a Jacobian **P** that causes maximal correlation between perceptual dissimilarity (computed between each $\overset{{}_{⇀}}{s}$ and $\overset{{}_{⇀}}{s}{}^{'}$ using Equation 16) and human difference ratings.

Approaches I and II will both be evaluated in the following sections.

## Methods

### Derivation of perceptual distance

In this paper, we defined $\overset{{}_{⇀}}{u}\left(\overset{{}_{⇀}}{s}\right)$ as the distortion field which places images $\overset{{}_{⇀}}{s}$ in new locations ${\overset{{}_{⇀}}{s}}_{P}$ where, locally, perceived difference matches Euclidean distance. We wrote that Equation 2, $d_{E}^{2}\left(\overset{{}_{⇀}}{s},\overset{{}_{⇀}}{s}{}^{'}\right)={\left(\overset{{}_{⇀}}{s}{}^{'}-\overset{{}_{⇀}}{s}\right)}^{T}\left(\overset{{}_{⇀}}{s}{}^{'}-\overset{{}_{⇀}}{s}\right)$, can be meaningfully converted into Equation 9, $d_{P}^{2}\left(\overset{{}_{⇀}}{s},\overset{{}_{⇀}}{s}{}^{'}\right)={\left(\overset{{}_{⇀}}{s}{}^{'}-\overset{{}_{⇀}}{s}\right)}^{T}\left(I+{\overset{{}_{⇀}}{\nabla }}_{s}{\overset{{}_{⇀}}{u}}^{T}\right)I\left(I+{\left({\overset{{}_{⇀}}{\nabla }}_{s}{\overset{{}_{⇀}}{u}}^{T}\right)}^{T}\right)\left(\overset{{}_{⇀}}{s}{}^{'}-\overset{{}_{⇀}}{s}\right)$. Let us derive this using Figure 2. The difference between perceptual image coordinates is given by Equation 4, $d_{P}^{2}\left(\overset{{}_{⇀}}{s},\overset{{}_{⇀}}{s}{}^{'}\right)={\left(\overset{{}_{⇀}}{s}{}^{'}\;{\;}_{P}-{\overset{{}_{⇀}}{s}}_{P}\right)}^{T}\;\left(\overset{{}_{⇀}}{s}{}^{'}\;{\;}_{P}-{\overset{{}_{⇀}}{s}}_{P}\right)$. By applying Equation 3 (or by using a little trigonometry on Figure 2):

(19) $$\begin{array}{ccc} d_{P}^{2}\left(\overset{{}_{⇀}}{s},\overset{{}_{⇀}}{s}{}^{'}\right) & = & {\left(\overset{{}_{⇀}}{s}{}^{'}+\overset{{}_{⇀}}{u}\left(\overset{{}_{⇀}}{s}\;\;{}^{'}\right)-\overset{{}_{⇀}}{s}-\overset{{}_{⇀}}{u}\left(\overset{{}_{⇀}}{s}\right)\right)}^{T}\\ & & \times \left(\overset{{}_{⇀}}{s}{}^{'}+\overset{{}_{⇀}}{u}\left(\overset{{}_{⇀}}{s}\;\;{}^{'}\right)-\overset{{}_{⇀}}{s}-\overset{{}_{⇀}}{u}\left(\overset{{}_{⇀}}{s}\right)\right)\; \end{array}$$

Importantly, we can replace $\overset{{}_{⇀}}{u}\left(\overset{{}_{⇀}}{s}\;\;{}^{'}\right)$ with $\overset{{}_{⇀}}{u}\left(\overset{{}_{⇀}}{s}\right)$ *plus* the degree to which $\overset{{}_{⇀}}{u}\left(\overset{{}_{⇀}}{s}\;\;{}^{'}\right)$ differs from $\overset{{}_{⇀}}{u}\left(\overset{{}_{⇀}}{s}\right)$ as we move from $\overset{{}_{⇀}}{s}$ to $\overset{{}_{⇀}}{s}\;\;{}^{'}$:

(20) $$\begin{array}{c} \overset{{}_{⇀}}{u}\left(\overset{{}_{⇀}}{s}\;\;{}^{'}\right)=\overset{{}_{⇀}}{u}\left(\overset{{}_{⇀}}{s}\right)+{\left({\overset{{}_{⇀}}{\nabla }}_{s}{\overset{{}_{⇀}}{u}}^{T}\right)}^{T}\left(\overset{{}_{⇀}}{s}{}^{'}-\overset{{}_{⇀}}{s}\right)\; \end{array}$$

We apply this replacement to Equation 19, then cancel the $\overset{{}_{⇀}}{u}\left(\overset{{}_{⇀}}{s}\right)-\overset{{}_{⇀}}{u}\left(\overset{{}_{⇀}}{s}\right)$ terms. This yields a function only of images and changes to $\overset{{}_{⇀}}{u}$ as a function of changes to pixel intensity:

(21) $$\begin{array}{ccc} \;d_{P}^{2}\left(\overset{{}_{⇀}}{s},\overset{{}_{⇀}}{s}{}^{'}\right) & = & {\left(\overset{{}_{⇀}}{s}{}^{'}+{\left({\overset{{}_{⇀}}{\nabla }}_{s}{\overset{{}_{⇀}}{u}}^{T}\right)}^{T}\left(\overset{{}_{⇀}}{s}{}^{'}-\overset{{}_{⇀}}{s}\right)-\overset{{}_{⇀}}{s}\right)}^{T}\\ & & \;\times \left(\overset{{}_{⇀}}{s}{}^{'}+{\left({\overset{{}_{⇀}}{\nabla }}_{s}{\overset{{}_{⇀}}{u}}^{T}\right)}^{T}\left(\overset{{}_{⇀}}{s}{}^{'}-\overset{{}_{⇀}}{s}\right)-\overset{{}_{⇀}}{s}\right)\; \end{array}$$

We reorder this equation and then distribute the outer transpose:

(22) $$\begin{array}{ccc} \;d_{P}^{2}\left(\overset{{}_{⇀}}{s},\overset{{}_{⇀}}{s}{}^{'}\right) & = & \left({\left(\overset{{}_{⇀}}{s}{}^{'}-\overset{{}_{⇀}}{s}\right)}^{T}+{\left(\overset{{}_{⇀}}{s}{}^{'}-\overset{{}_{⇀}}{s}\right)}^{T}\;{\overset{{}_{⇀}}{\nabla }}_{s}{\overset{{}_{⇀}}{u}}^{T}\right)\\ & & \;\times \left(\left(\overset{{}_{⇀}}{s}{}^{'}-\overset{{}_{⇀}}{s}\right)+{\left({\overset{{}_{⇀}}{\nabla }}_{s}{\overset{{}_{⇀}}{u}}^{T}\right)}^{T}\left(\overset{{}_{⇀}}{s}{}^{'}-\overset{{}_{⇀}}{s}\right)\right)\; \end{array}$$

Next, we factor each half of the formula, returning the equation to a form we recognize as equivalent to Equation 9:

(23) $$\begin{array}{ccc} \;d_{P}^{2}\left(\overset{{}_{⇀}}{s},\overset{{}_{⇀}}{s}{}^{'}\right) & = & {\left(\overset{{}_{⇀}}{s}{}^{'}-\overset{{}_{⇀}}{s}\right)}^{T}\left(\;I+{\overset{{}_{⇀}}{\nabla }}_{s}{\overset{{}_{⇀}}{u}}^{T}\;\right)I\\ & & \;\times \left(\;I+{\left({\overset{{}_{⇀}}{\nabla }}_{s}{\overset{{}_{⇀}}{u}}^{T}\right)}^{T}\;\right)\left(\overset{{}_{⇀}}{s}{}^{'}-\overset{{}_{⇀}}{s}\right)\; \end{array}$$

We started with a simple difference measure in terms of unknown perceptual/neural representations, ${\left(\overset{{}_{⇀}}{s}{}^{'}\;{\;}_{P}-{\overset{{}_{⇀}}{s}}_{P}\right)}^{T}\;\left(\overset{{}_{⇀}}{s}{}^{'}\;{\;}_{P}-{\overset{{}_{⇀}}{s}}_{P}\right)$. Equation 23 looks somewhat like the equations that Pons and colleagues (1999, equation 18) and Laparra and colleagues (2010, equation 11) used to construct their nonlinear perceptual metrics. This equation has now been converted into a strained difference measure in terms of image pixels.

### Derivation of strain tensor

In the previous subsection, we could have replaced Equation 9 with the inner multiplication:

(24) $$\begin{array}{ccc} d_{P}^{2}\left(\overset{{}_{⇀}}{s},\overset{{}_{⇀}}{s}{}^{'}\right) & = & {\left(\overset{{}_{⇀}}{s}{}^{'}-\overset{{}_{⇀}}{s}\right)}^{T}(I+{\overset{{}_{⇀}}{\nabla }}_{s}{\overset{{}_{⇀}}{u}}^{T}+\;{\left({\overset{{}_{⇀}}{\nabla }}_{s}{\overset{{}_{⇀}}{u}}^{T}\right)}^{T}\\ & & +{\overset{{}_{⇀}}{\nabla }}_{s}{\overset{{}_{⇀}}{u}}^{T}{\left({\overset{{}_{⇀}}{\nabla }}_{s}{\overset{{}_{⇀}}{u}}^{T}\right)}^{T})\left(\overset{{}_{⇀}}{s}{}^{'}-\overset{{}_{⇀}}{s}\right) \end{array}$$

The last term in the middle is an order smaller than the other terms. If we assume that it is vanishingly small, we reach a new equation:

(25) $$\begin{array}{ccc} \;d_{P}^{2}\left(\overset{{}_{⇀}}{s},\overset{{}_{⇀}}{s}{}^{'}\right) & = & {\left(\overset{{}_{⇀}}{s}{}^{'}-\overset{{}_{⇀}}{s}\right)}^{T}\left(I+{\overset{{}_{⇀}}{\nabla }}_{s}{\overset{{}_{⇀}}{u}}^{T}+{\left({\overset{{}_{⇀}}{\nabla }}_{s}{\overset{{}_{⇀}}{u}}^{T}\right)}^{T}\right)\\ \; & & \times \left(\overset{{}_{⇀}}{s}{}^{'}-\overset{{}_{⇀}}{s}\right) \end{array}$$

We define the strain tensor based on the above equation:

(26) $$\begin{array}{c} d_{P}^{2}\left(\overset{{}_{⇀}}{s},\overset{{}_{⇀}}{s}{}^{'}\right)={\left(\overset{{}_{⇀}}{s}{}^{'}-\overset{{}_{⇀}}{s}\right)}^{T}\left(I+2ɛ\right)\left(\overset{{}_{⇀}}{s}{}^{'}-\overset{{}_{⇀}}{s}\right)\; \end{array}$$

where **ε** is the strain tensor and **I**, the identity matrix, was the original tensor. By distribution of the previous equation, we can create an alternative definition of perceptual distance (not required herein):

(27) $$\begin{array}{c} d_{P}^{2}\left(\overset{{}_{⇀}}{s},\overset{{}_{⇀}}{s}{}^{'}\right)\to d_{E}^{2}\left(\overset{{}_{⇀}}{s},\overset{{}_{⇀}}{s}{}^{'}\right)+2\;{\left(d\overset{{}_{⇀}}{s}\right)}^{T}ɛd\overset{{}_{⇀}}{s}\; \end{array}$$

where $2{\left(d\overset{{}_{⇀}}{s}\right)}^{T}ɛd\overset{{}_{⇀}}{s}$ is the *change in distance* caused by perceptual strain.

### Published datasets

We evaluate our perceptual model on three industry-standard datasets. The Categorical Subjective Image Quality (CSIQ) dataset (Larson & Chandler, 2010) contains 30 hand-selected color 512 × 512 pixel images of animals, landscapes, people, plants, and urban scenes. The images were degraded to five different levels of JPEG fidelity, which human subjects (*N* < 35, precise count unknown) placed together on a linear scale such that pairwise distances between the images matched perceived difference. The TID 2013 (Ponomarenko et al., 2015) and Toyama (Tourancheau, Autrusseau, Sazzad, & Horita, 2008) datasets were also utilized for breadth. These datasets contain similar imagery, with slightly varying image sizes and measures. Each of these datasets contains subsets with different image degradation methods (see citations). Regardless of dataset, all human ratings reported here are normalized to a range of [0, 1], where 0 is no perceived distance (perfect fidelity).

Preexisting datasets have been shown to be inconsistent benchmarks, even when datasets contain almost the exact same images. CSIQ, TID 2013, and Toyama share many images but prefer different IQA measures (Lin & Kuo, 2011; Winkler, 2012). Our work also raises the possibility of comparing natural image statistics with perceptual geometry. Such comparisons would require more plentiful imagery to overcome natural image variability and a more targeted set of natural scenes, known to vary in image statistics. We therefore introduce the new Scene Image Quality (SceneIQ) dataset, based on reference images with well-characterized statistics (Oliva & Torralba, 2001). Whereas CSIQ, TID 2013, and Toyama each contains less than 50 original (non-degraded) images, SceneIQ contains 2080 original images.

### Newly acquired sceneIQ dataset

**Figure 4.**
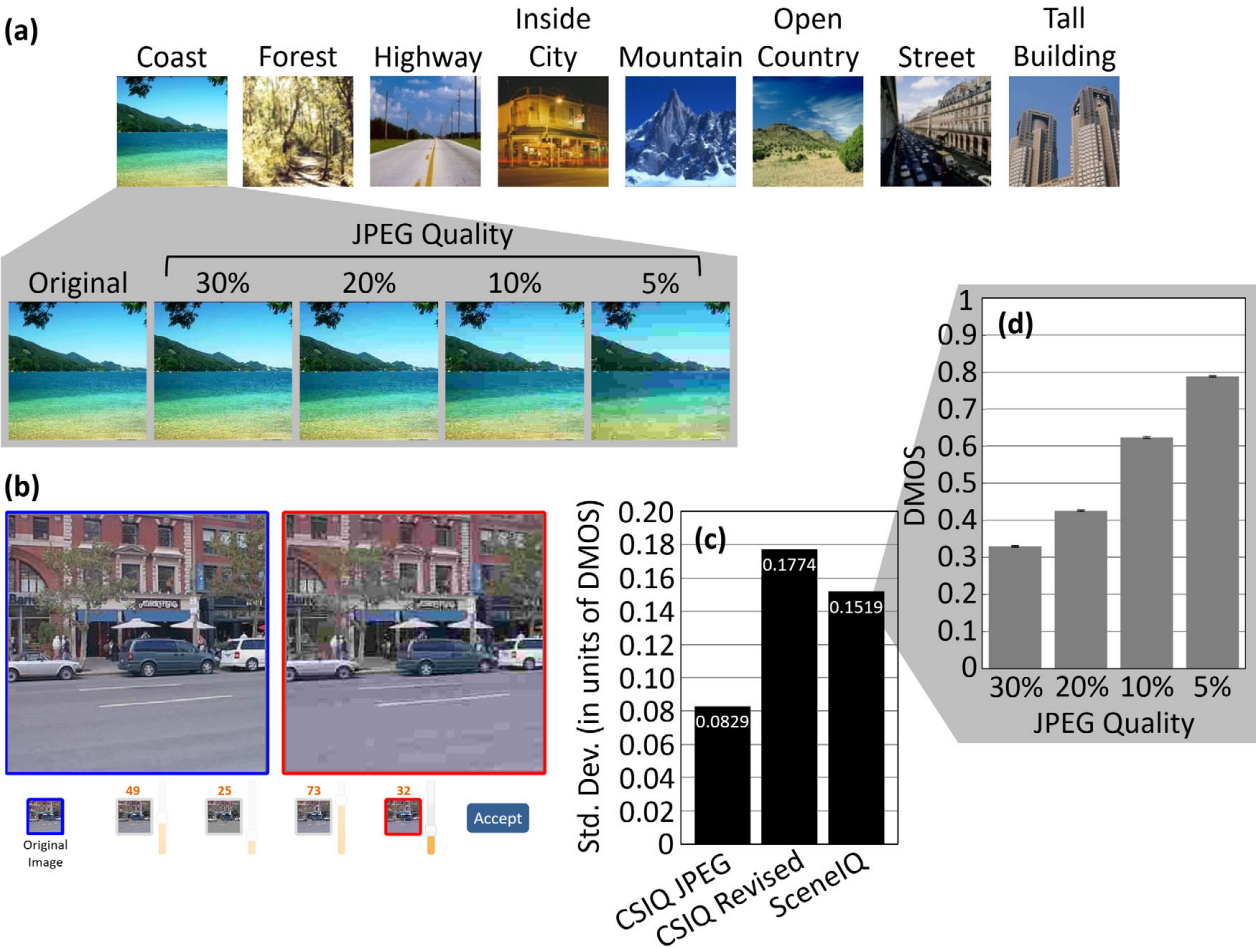
Characteristics of the SceneIQ dataset. (a) Example images. (b) Layout of the experimental paradigm. (c) Mean of the subject-wise standard deviation across all images and quality levels for three sets of data (left to right): the CSIQ (JPEG) dataset's original DMOS scores, DMOS scores for CSIQ non-degraded images degraded at the same quality levels used on the SceneIQ dataset and scored on MTurk, and the SceneIQ scores. (d) SceneIQ Online dataset. Mean DMOS score increased as the JPEG quality decreased (humans rate lower fidelity images as being lower fidelity). Bars are standard error across images (*N* = 2080).

We acquired human fidelity ratings for a public set of 256 × 256 pixel color images (Oliva & Torralba, 2001), split into eight scene categories: seacoast, forest, highway, inside city, mountain, open country, street, and tall building (for examples, see Figure 4a). The images were randomly subsetted from the original publication to equalize *N* across categories. We used 260 images per category, the number of images in the rarest category. Each image in the dataset was degraded into four JPEG quality levels: 30%, 20%, 10%, and 5% using ImageJ (National Institutes of Health, Bethesda, MD) (Schneider, Rasband, & Eliceiri, 2012).

The dataset contains 260 × 8 = 2080 non-degraded images and 8320 degraded images. This high count is important to reduce regression overfitting and evaluate higher order statistics but poses a problem because no single subject can rate this many images. Although other assignment strategies were evaluated, we decided to split the subjects into groups. Each subject was randomly assigned to a set of 40 images, without replacement, such that every image is seen exactly once within one group of 52 people. We collected enough data for five groups, or 260 subjects from (primarily) American humans on Amazon Mechanical Turk (MTurk). Each image was seen by five people, yielding a total of 41,600 ratings. Although human ratings were based on color JPEGs, all IQA algorithms used grayscaled versions. This is the standard procedure in the field of IQA—measures such as SSIM cannot be computed on multispectral data. For all computer IQA measures, images were normalized (luminance stretched) to a range of 0 to 255.

In each randomly shuffled trial, one non-degraded image was presented per screen, along with the four degraded versions of the same image in random order. To make a series of pairwise comparisons, subjects could left click to magnify any thumbnail in the left box and right click to magnify it in the right box (screenshot in Figure 4b). Subjects were instructed to rate each degraded image based on how different it was from the original, on an integer scale from 0 to 100 (instructions are available in Supplementary Figure S1). These ratings were converted to a difference mean opinion score (DMOS, a standardized measure) by the equation DMOS = 1 – (rating/100). A DMOS of 1 rates two images as 100% different (an undefined concept). A DMOS of 0 indicates that images have no perceptible difference. When subjects were satisfied with the correctness of all four ratings (without time constraint), they clicked “Accept” to advance to the next trial.

Ratings obtained via MTurk involve everyday viewing conditions and thus are less controlled for viewing parameters. So, to validate/baseline these SceneIQ Online DMOS ratings obtained via MTurk, we compared them with the commonly accepted DMOS scores of CSIQ. As indicated by Figure 4c, the original DMOS scores for the CSIQ dataset, collected in a controlled environment, have on average half the variance of those collected using our online paradigm. However, online data collection yielded data with more subjects and more images. More data reduces standard error and enhances power (indeed, Figure 4d indicates small standard error bars in one simple analysis). This approach has been shown to improve statistical significance in unrelated work (Buhrmester, Kwang, & Gosling, 2011). Perhaps, the ecological validity of real, variable viewing conditions makes these ratings an even more reliable benchmark than scores collected under highly controlled conditions.

Five subjects were discarded and replaced with new ones. Two were discarded because their data became corrupted during collection, two were discarded because they self-reported as having poor vision, and one was discarded for disregarding task instructions, almost always responding with the maximum rating value. To avoid biasing the dataset, we chose liberal inclusion criteria. To enable more strict exclusion of subjects that poorly adhered to the task, future iterations of this dataset would benefit from catch trials or measures of task performance orthogonal to the dependent variables.

The paradigm and stimuli were replicated in a controlled laboratory setting at Dartmouth (SceneIQ Lab dataset). All subjects used the same high-quality screen (U28D590; Samsung, Seoul, South Korea), same private viewing room, and similar viewing distance (50.8 cm, 20 inches). Forty-nine subjects (18–22 years old; 35 females) participated in rating degraded versions of 600 original (non-degraded) images (75 per semantic category), extracted from the same image source as SceneIQ (Oliva & Torralba, 2001) and degraded identically. Four participants were discarded for incomplete data.

To eliminate uncontrolled inconsistencies between the CSIQ and SceneIQ datasets, we will also report a dataset (CSIQ Revised) collected using CSIQ original (non-degraded) images but SceneIQ image degradation and methodology. Consistently with SceneIQ, we degraded each CSIQ image to four JPEG quality levels: 30%, 20%, 10%, and 5% using ImageJ (Schneider et al., 2012). Forty subjects each rated all images via MTurk using the same paradigm as SceneIQ Online. No subjects were discarded.

All human studies were approved by the Dartmouth institutional review board. Additional summary statistics are visible in Supplementary Figure S2. SceneIQ can be acquired online at https://github.com/DartmouthGrangerLab/SceneIQ or by contacting the authors. Additional methodological details can also be found online.

### Approach I

The human perceptual operator was first described by a Gaussian function with a standard deviation of σ. Only the SceneIQ Lab dataset contains the viewing parameters needed to convert our stimulus model in units of pixels (px) into degrees visual angle (°). Therefore, we present all models in terms of pixels, and approximate degrees visual angle for all datasets based on the SceneIQ Lab viewing parameters. For SceneIQ Lab, the center pixel was 0.0619°. Given the Gaussian hypothesis, we seek the optimal parameterization of σ and judge the hypothesis on its best parameterization. (Prior works have similarly optimized hypothesis parameters using real data; see Wang et al., 2003; Wu, Lin, Shi, & Liu, 2013). The model was evaluated with σ in the range [0.4, 3.0] px, or [0.0247, 0.1857]°, by increments of 0.1 px (0.0062°). Across five random folds of the original (non-degraded) images, we recorded the σ of the highest correlations (minimum error) with DMOS.

The perceptual operation was next described by a difference between two concentric Gaussian functions. We evaluated a range of values for center Gaussian width by increments of 0.2 px (0.0124°) on the range [0.6, 5.0] px ([0.0371, 0.3095]°); surround (negative) Gaussian width in increments of 0.2 px (0.0124°) on the range [0.6, 5.6] px ([0.0371, 0.3466]°) (larger values become slow to compute); and the ratio of maximum heights between the two Gaussians, α (increments of 0.1 px (0.0062°), range [0.5,1.5] px ([0.0310,0.0929]°). Using the same cross validation procedure as before, we recorded Pearson correlations with SceneIQ Online DMOS for each possible parameter combination.

Both connectivity patterns described in this section were derived from biology. However, their parameterizations were derived by model fit. This yields a high-performing hybrid Jacobian but does not fully evaluate the relative contributions of actual Gaussian and difference-of-Gaussians connectivity profiles in the human. We look forward to further evaluations of approach I with stimulus-independent, biologically derived Gaussian widths.

### Approach II

**Figure 5.**
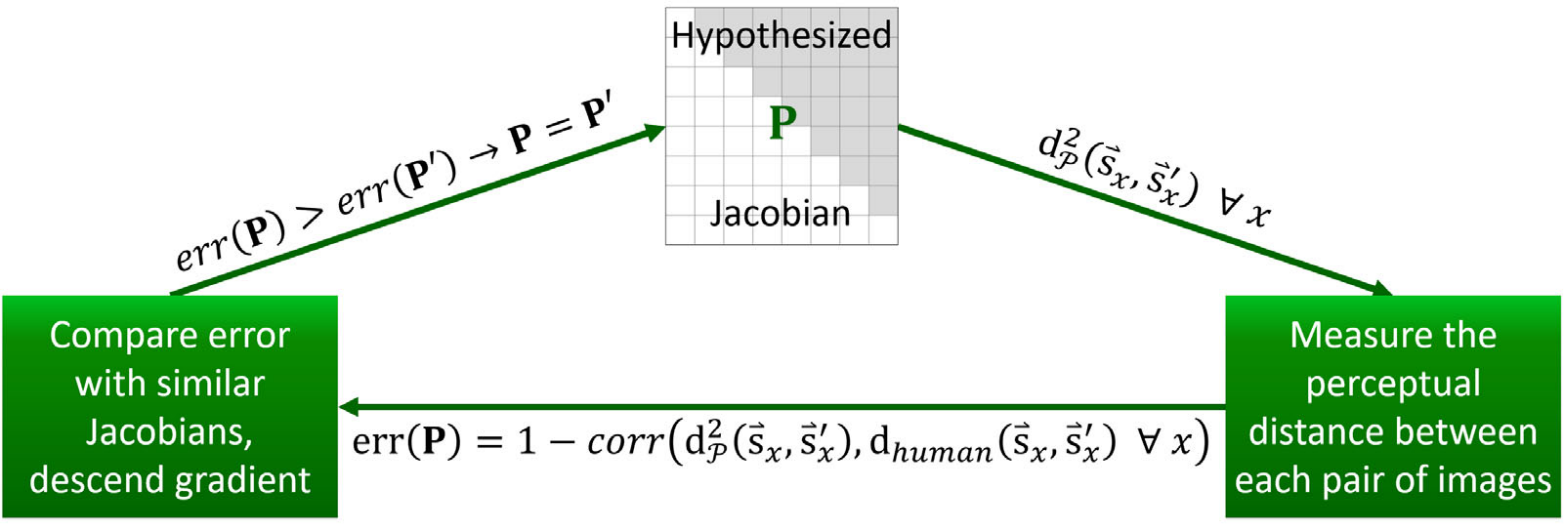
Determination of an approach II Jacobian by way of regression. A Jacobian is initialized. Second, the Jacobian is used to measure the distance between each image pair. Third, the error of this Jacobian is computed based on how well its distances correlate with human subject ratings. If this Jacobian produces reduced error, it is marked as the best working hypothesis. Finally, new Jacobians are generated with slight deviations from the best working hypothesis.

Our second approach uses regression to find a perceptual Jacobian that measures the distance between image pairs ($\overset{{}_{⇀}}{s}{}^{'}-\overset{{}_{⇀}}{s}$) in a humanlike way—in correlation with DMOS scores. Treating our regression as an optimization problem, we performed random walk gradient descent on the set of all possible combinations of values for the cells of the Jacobian. The Jacobian was initialized to the identity matrix (the initial distance measure was Euclidean). At each iteration, the algorithm randomly selected a cell of the Jacobian. This corresponded to a particular dimension of the error surface. The algorithm then evaluated the error of the Jacobian with this cell increased by 0.1 and the error of the Jacobian with this cell decreased by 0.1. Error was defined as 1 − Pearson correlation between the DMOS scores for all training images and the distance scores provided by the new Jacobian. If either modified Jacobian caused error to be reduced, the evaluated Jacobian with the smallest error was chosen as the new Jacobian. The algorithm iterated for 10,000 steps, which we subjectively determined to be the point at which error plateaued (a minimum was found; see Supplementary Figure S10). This algorithm is visualized in Figure 5. Approach II succeeds despite the extreme simplicity of its optimization algorithm, which we find to be an argument for the power of the general approach. Regularization was avoided because it would be difficult to interpret the resultant Jacobian if its values are attributed to an unknown combination of correctness and regularization terms (e.g., sparsity).

In order to more easily interpret these results, we make the limiting assumption for approach II that a single Jacobian is applicable across the set of all images (or all examples of a scene category). Because this Jacobian is nonspecific to a particular type of image, we will be extracting only those perceptual components applicable to all images. Several formalizations of perceptual geometry in the literature (Epifanio et al., 2003; Laparra et al., 2010; Malo et al., 2000; Malo et al., 2005; Pons et al., 1999) offer hints at the potential of a future “approach III” capable of constructing highly signal-dependent Jacobians. Work remains to be done to generalize this biological connectivity approach to situations in which perceptual strain is highly variable.

For efficiency and because the Jacobian must be a symmetric matrix to guarantee that the tensor will fulfill the desirable property of symmetry, the upper diagonal of the Jacobian is dependent only on the lower triangle. The diagonal is fixed to ones, accounting for the identity matrix in Equation 15. Therefore, the error surface is $\frac{D\;\times \;(D-1)}{2}$ dimensional. Cells of the Jacobian were limited to the range [−1, 1].

For the sake of computational complexity, we assume for approach II that **P** is local and uniform across the image (see Discussion for a simple relief from this extension). This assumption is congruent with the low-level visual system, wherein relations between representations of topographical neighbors are one dominant component (Dacey et al., 2000; De Monasterio, 1978; Hubel & Wiesel, 1962; Martinez & Alonso, 2003; Sincich & Blasdel, 2001; Von der Malsburg, 1973; Young, 1987; Young & Lesperance, 2001). It is also approximately true for the radial basis functions explored in approach I. Images were split into 8 × 8-pixel tiles. This enabled us to optimize a single 64 × 64 Jacobian (rather than a 65,536 × 65,536 Jacobian that has an untenable billion-dimensional error surface). The 64 × 64 Jacobian was used to compare each tile of an image, after which the tile distances were summed. This tiling approach is consistent with JPEG (Pennebaker & Mitchell, 1992; Wallace, 1992), related compression methods (Bowen, Felch, Granger, & Rodriguez, 2018), and other IQA measures (e.g., SSIM; Wang et al., 2004). Sub-imaging greatly increased the number of data points used for training while simplifying the task.

Several considerations are relevant to this regression. We present results using a regression that allowed cells of the Jacobian to be negative. Negative cells in the Jacobian indicate that certain features contradict one another. Anecdotally, we found these results to be superior to non-negative regressions. Second, the solution of a gradient descent optimization may not be unique (the regression may find one of many local optima). Normally, researchers can find the global optimum by performing multiple regressions with different randomly initialized Jacobians. However, the high dimensionality of the error space means that it cannot be sufficiently sampled in a reasonable amount of time, so it is not naïvely practical to find a global minimum. We make an anecdotal report that different random regressions (although all starting from the identity matrix) produce nearly identical results. This is likely because the implicit dimensionality of natural images is low (Seung & Lee, 2000; Simoncelli & Olshausen, 2001; Torralba & Oliva, 2003), as most regions of image space are unpopulated.

### Analysis

Models were evaluated by measuring the Pearson correlations between model predictions and human DMOS ratings, in log–log coordinates. We have found that the relationship between human ratings and model predictions is often clearer in log–log space. We want to measure the statistical significance of pairwise differences between correlations with DMOS for competing models. One option is to perform X-fold cross validation of the original (non-degraded) images and measure the significance across folds of the difference between two models. Due to the regression runtime for approach II, we can at most acquire five folds. Five pairs is too few for the nonparametric Wilcoxon signed-rank test or Fisher–Pitman exact permutation test—the minimum possible *p* value is not significant. By contrast, the paired *t*-test (on Fisher *z*-transformed correlations) can yield any *p* value but will be sensitive to outliers and variability in the results. Instead, we measured, for each of the two folds reported in Table 1, a two-tailed Fisher *r*-to-*z* transformation, then took the mean across folds.

For within-dataset analyses, the training set of original images was randomly halved (preserving equal *N* among semantic categories), and two approach II Jacobians were independently optimized in a two-fold cross validation. Each Jacobian was only used to predict images uninvolved in its training, and the two sets of test scores were pooled (without modification) for comparison with DMOS.

For across-dataset analyses, an approach II Jacobian was optimized on one entire dataset (e.g., SceneIQ Lab) and then used to predict the mean subject's DMOS score for each image of a separate dataset.

## Results and predictive capacity

The question asked is do the tensors produced by approaches I and II explain most of the variance in human ratings not already explained by Euclidean measures? To test the ability of each connectivity pattern to predict human similarity judgments, we compared correlation with human ratings. The outcomes of these analyses are apparent in Table 1. In correlation with human ratings, Pearson's *r* = 0.45 for Euclidean (MSE); *r* = 0.63 for approach I Gaussian σ = 0.6 px (0.0310°); *r* = 0.83 for approach I DOG; and *r* = 0.76 for approach II (SceneIQ Online, linear axes, mean across two folds of data) all differ from chance (*p* << 0.001) (Table 1). Comparisons in terms of rank-order correlation and logistic regression are included in Supplementary Materials.

**Figure 6.**
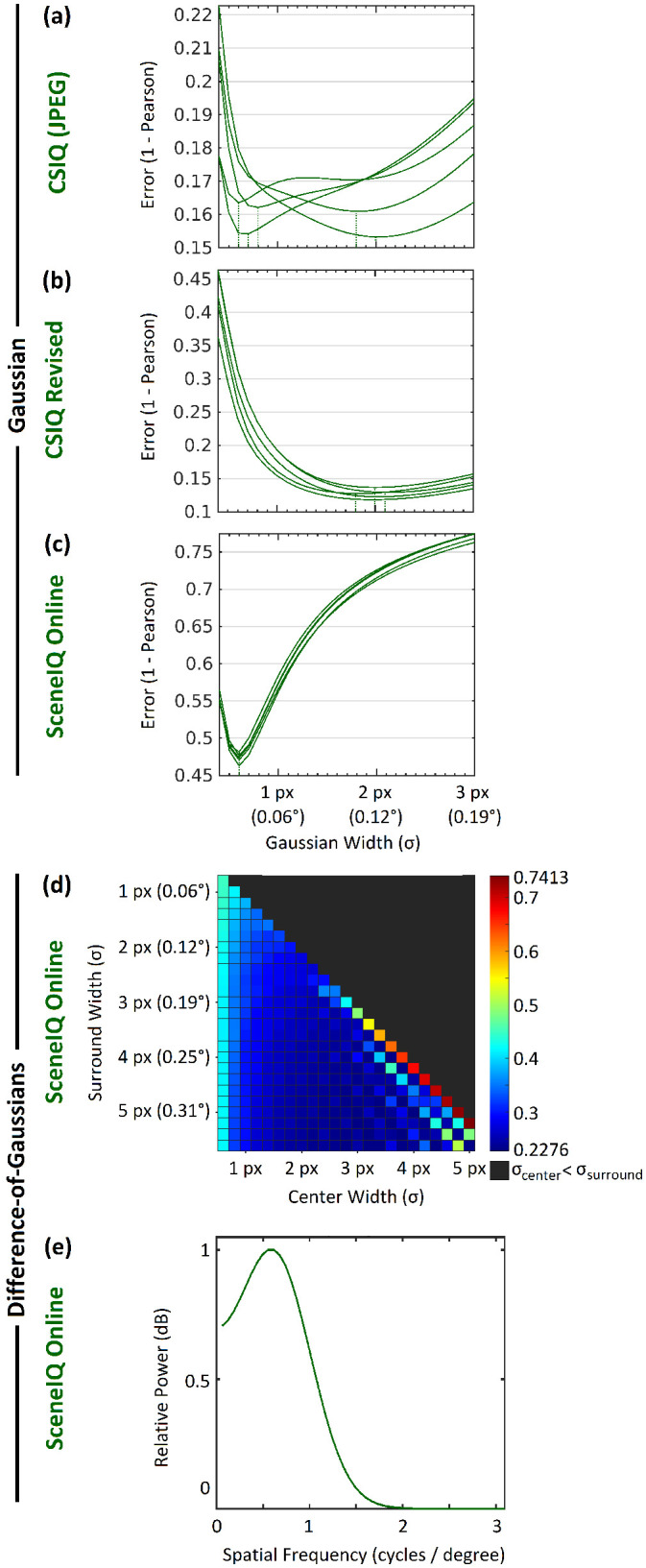
Approach I optimality with various Gaussian widths. A range of Gaussian widths (σ) was evaluated for each of five random folds of the (a) CSIQ (JPEG) dataset, (b) CSIQ Revised dataset, and (c) SceneIQ Online dataset using Pearson correlation. Dashed lines mark the global minima of each fold. (d) Difference-of-Gaussians training error as a combined function of σ_center_ and σ_surround_, on SceneIQ Online fold 1 of 2. For visualization, the third parameter (α) was eliminated by selecting its optimal value for each combination of σ_center_ and σ_surround_. (e) Contrast sensitivity function computed from the best difference-of-Gaussians parameters (see text).

Error curves for approach I Gaussians of various widths are compared in Figure 6. We present further analyses using two parameters, σ = 2.0 px (0.1238°) for CSIQ Revised and σ = 0.6 px (0.0310°) for SceneIQ, found by taking the mean of the minimum-error σ across folds (rounded to 0.1). We did not select a parameter from CSIQ (JPEG) due to high variance in the minimum-error σ across folds. We did not note at the time that there may be some similarity between these values and the correlation among pixels recorded in the image dataset itself (see Supplementary Figure S9b).

Figure 6d reports the two-dimensional error curve for difference-of-Gaussians models on one fold of the SceneIQ Online dataset. All folds reported the same local maximum: σ_center_ = 3.6 px (0.2228°), σ_surround_ = 5.2 px (0.3219°), α = 0.7. In comparison with neuronal response profiles (available for visualization in Figure 3), these parameters appear intermediate: More broad profiles have been found in retinal ganglion cells (Dacey, 1996; Dacey, 2000; Rodieck, 1965). More narrow and Gaussian-like profiles have been found by others (Croner & Kaplan, 1995; Dacey et al., 2000). From this DOG parameterization and the equation for DOG(*x*) in Equation 12, we can compute the contrast sensitivity function (CSF) that these parameters hypothesize (Wandell, 1995):

(28) $$\begin{array}{c} \begin{array}{c} y_{i}=DOG\left(i\right)/DOG\left(0\right)\forall i\in \left\{-128..128\right\}\\ \overset{{}_{⇀}}{z}\;=fft\left(\overset{{}_{⇀}}{y}\right)\\ z_{i}=\left(2/257\right)z_{i}^{2}\forall i\in \left\{2..129\right\} \end{array}\; \end{array}$$

The result is depicted in Figure 6e in terms of decibels, where *z_i_* = 10 log_10_(1 + *z_i_*). The shape of this CSF is similar to those computed from DOG parameters in other works. For example, Wuerger, Watson, and Ahumada (2002) fit difference-of-Gaussians parameters to human behavioral responses. The authors used these parameters as the basis of a spatial luminance CSF. In visual crowding, it was recently found that a novel measure of contrast, capable of relating DOG parameters to contrast sensitivity, accounts for a substantial amount of data (Rodriguez & Granger, 2021).

This CSF peaks at 0.56 cycles per degree. Unlike CSFs found by some Gabor-based approaches (Chandler, 2013; Dacey, 2000), its shape hints at a bandpass (vs. lowpass) CSF. We note that CSFs have been used directly in many measures of image quality. Li, Lu, Tao, and Gao (2008) acquired a bandpass CSF from prior experimentation (Mannos & Sakrison, 1974), then applied it as a mask on the wavelet-transformed image to enhance SSIM. However, this CSF peaks drastically earlier (0.56 vs. 8 cycles per degree). The peak of our CSF is more comparable with those computed from difference-of-Gaussians luminance models by Wuerger and colleagues (2002). Still, the peak of this CSF is low by human standards (Souza, Gomes, & Silveira, 2011), perhaps hinting that subjects viewed the images peripherally.

**Figure 7.**
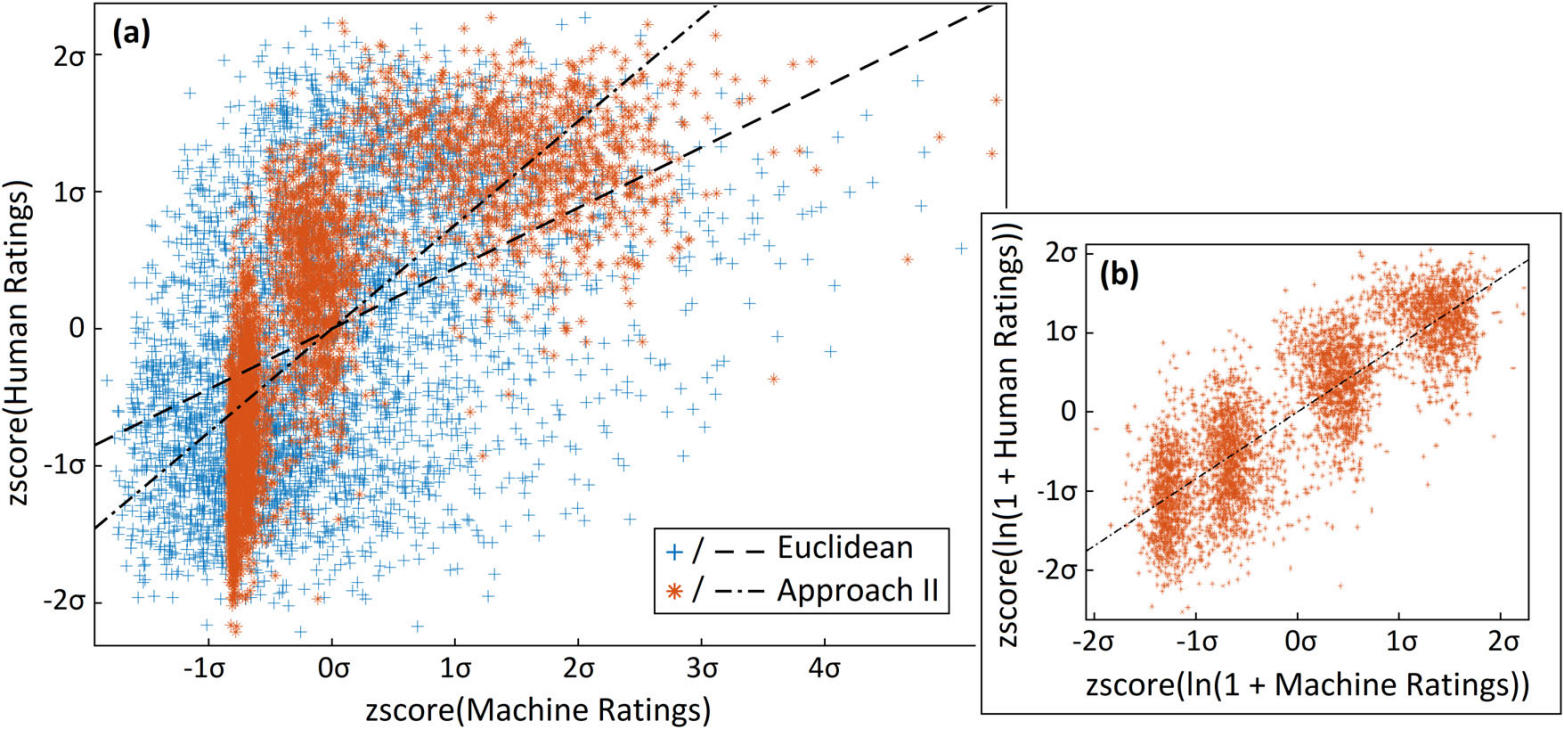
Correlation of Euclidean and approach II with DMOS. Half (first fold) of the full SceneIQ Online dataset. Machine ratings are on the *x*-axis, and human DMOS ratings are on the *y*-axis. We have plotted lines of best fit. (a) Pearson's correlation: Euclidean *r* = 0.44; approach II *r* = 0.76. Euclidean and approach II ratings were *z*-scored separately so they could be more usefully superimposed. (b) Approach II against human ratings, reproduced on log–log axes. Pearson's correlation on these axes was *r* = 0.85. Logistic fits and comparisons with SSIM are available in Supplementary Materials.

It is important that the relationship between predictions and behavior be simple. Complicated relationships (e.g., logistic fits used in many IQA methods) or those with many parameters require further explanation. Figure 7a illustrates empirical DMOS scores from SceneIQ Online as predicted by Euclidean and approach II. In this case, the fit between approach II and empirical human ratings (DMOS) appears linear when plotted on log–log axes (Figure 7b). In Figure 7, the four groupings of approach II ratings roughly correspond to the four JPEG quality levels in the dataset.

We compare the predictive capacity of approaches I and II against performance-driven IQA measures from the literature: SSIM, MS-SSIM (Wang et al., 2003), IWSSIM (Wang & Li, 2011), VSNR (Chandler & Hemami, 2007), VIF (Sheikh & Bovik, 2006), VIFP (Sheikh & Bovik, 2006), IFC (Sheikh, Bovik, & De Veciana, 2005), and GMSD (Xue et al., 2014). Table 2 compares these machine rating measures with one another, depicting significant differences in performance.

**Table 2.** Differences among Pearson's *r*. Entries in this table indicate the probability that the proposed models (columns) and alternative models (rows) correlate equally with humans on SceneIQ Online (all). The *p* values were determined by two-tailed Fisher *r*-to-*z* transformation of Pearson correlations (as measured for each of two folds, then meaned; unadjusted). ^*^Indicates values below conservative Bonferroni thresholds for 28 comparisons at 0.05 (28 comparisons per proposed model/column).

	Gaussian σ = 0.6 px (0.0310°)	Gaussian σ = 2 px (0.1238°)	Center surround (DOG)	Approach II SceneIQ Online
On log–log axes				
Euclidean (MSE)	0^*^	0^*^	0^*^	0^*^
SSIM	>0.3	0^*^	0^*^	0^*^
MS-SSIM	0^*^	0^*^	0^*^	0^*^
IWSSIM	0^*^	0^*^	0^*^	0^*^
VSNR	>0.01	0^*^	0^*^	0^*^
VIF	0^*^	0^*^	>0.007	>0.2
VIFP	0^*^	0^*^	0^*^	0^*^
IFC	0^*^	0^*^	0^*^	0^*^
GMSD	0^*^	0^*^	>0.0002^*^	>0.02
PerceptNet	0^*^	0^*^	0^*^	0^*^
BioMultilayer	0^*^	0^*^	0^*^	0^*^
Gaussian σ = 0.6 px (0.0310°)	1	>0.000001^*^	0^*^	0^*^
Gaussian σ = 2 px (0.1238°)	>0.000001^*^	1	0^*^	0^*^
Center surround (DOG)	0^*^	0^*^	1	>0.1
Approach II SceneIQ Online	0^*^	0^*^	>0.1	1
On linear axes				
Euclidean (MSE)	0^*^	>0.01	0^*^	0^*^
SSIM	>0.3	0^*^	0^*^	0^*^
MS-SSIM	0^*^	0^*^	0^*^	>0.001^*^
IWSSIM	0^*^	0^*^	0^*^	>0.6
VSNR	>0.006	0^*^	0^*^	0
VIF	0^*^	0^*^	>0.0006^*^	>0.000003^*^
VIFP	0^*^	0^*^	0^*^	>0.4
IFC	>0.1	0^*^	0^*^	0^*^
GMSD	0^*^	0^*^	0^*^	0^*^
PerceptNet	0^*^	0^*^	0^*^	0^*^
BioMultilayer	0^*^	0^*^	0^*^	>0.3
Gaussian σ = 0.6 px (0.0310°)	1	0^*^	0^*^	0^*^
Gaussian σ = 2 px (0.1238°)	0^*^	1	0^*^	0^*^
Center surround (DOG)	0^*^	0^*^	1	0^*^
Approach II SceneIQ Online	0^*^	0^*^	0^*^	1

The above methods do not attempt to directly capture biological connectivity patterns nor link those to psychophysics—a central aim of this work. For this reason, we compare against two biologically relatable, shallow-layered, connectionist approaches nicknamed PerceptNet (Hepburn, Laparra, Malo, McConville, & Santos-Rodriguez, 2020) and BioMultilayer (Martinez-Garcia, Cyriac, Batard, Bertalmío, & Malo, 2018). BioMultilayer is of particular interest. Martinez-Garcia and colleagues (2018) decomposed the stimulus–response map into multiple successive sub-maps, each containing a nonlinear operator. Like our strain model, each of these operators constructs Jacobians to determine how the value of each feature (e.g., pixel) affects others. Unlike our approach, BioMultilayer directly utilizes psychophysical masking and divisive normalization.

SceneIQ is evenly divided across eight semantic categories of visual scene. For approaches I and II, a single Jacobian was identified for the set of all images. Nonetheless, static transforms are consistently performant psychophysical predictors across individual image categories (Table 1). They also can be seen to transfer well to the CSIQ dataset, where they remain among the best predictors. Unexpectedly, perception is easier to predict with this approach *across* scene categories than *within* them.

In Table 1, we highlight results on the JPEG degradation, because it is the same type used in the SceneIQ dataset. To evaluate the presented approaches in the broadest possible range of scenarios, we also evaluate results on other degradation types. Results on the “TID2013 Mean” contain the mean correlation across 23 degradation types (source data in Supplementary Table S5). To determine whether approaches I and II generalize across degradation methods, we measured correlations between model and human ratings for each of the five degradation types in the CSIQ dataset (Table 3). Although CSIQ is an unusually Euclidean-biased dataset, we find that approach II and approach I, to a lesser extent, generalize well to other degradation methods. We fit an approach II model to each CSIQ degradation method independently, then tested them on the same degradation using two-fold cross-validation and the same methodology as other approach II models. These models performed well but were generally outperformed by SceneIQ-fit models (possibly due to limited and noisy data).

**Table 3.** Pearson correlation with DMOS on CSIQ subsets. Pearson correlation with humans (DMOS) on log–log axes for the presented models (columns) on several datasets (rows). All values are calculated as the mean of two cross-validation folds of images. JP2K = jpeg2000 distortion, awgn = additive white gaussian noise. See text.

	CSIQ on log–log axes					CSIQ on linear axes				
	JPEG	JP2K	fnoise	blur	awgn	JPEG	JP2K	fnoise	blur	awgn
Approach I										
Gaussian σ = 0.6 px (0.0310°)	0.93	0.93	0.90	0.92	0.94	0.93	0.94	0.92	0.92	0.94
Gaussian σ = 2 px (0.1238°)	0.95	0.90	0.92	0.89	0.84	0.94	0.86	0.91	0.85	0.82
Center surround (DOG)	0.95	0.93	0.93	0.90	0.93	0.93	0.91	**0.92**	0.89	0.93
Approach II										
SceneIQ Online	0.94	**0.97**	**0.95**	**0.97**	**0.95**	0.77	0.84	0.85	0.82	0.89
SceneIQ Lab	0.94	0.97	0.95	0.97	0.95	0.77	0.84	0.85	0.82	0.89
CSIQ same degradation	0.89	0.94	0.95	0.93	0.95	0.88	0.78	0.85	0.78	0.89
Euclidean (MSE)	0.87	0.92	0.90	0.91	0.94	0.87	0.93	0.92	0.91	**0.94**
SSIM	0.86	0.81	0.73	0.79	0.80	0.84	0.78	0.72	0.76	0.74
MS-SSIM	0.86	0.81	0.71	0.80	0.79	0.87	0.82	0.71	0.81	0.79
IWSSIM	0.86	0.79	0.66	0.80	0.74	0.87	0.80	0.66	0.81	0.75
VSNR	0.88	0.87	0.84	0.82	0.90	0.88	0.88	0.85	0.83	0.90
VIF	**0.96**	0.92	0.91	0.89	0.95	0.90	0.72	0.89	0.73	0.94
VIFP	0.95	0.93	0.91	0.91	0.95	0.91	0.81	0.89	0.79	0.93
IFC	0.87	0.84	0.67	0.84	0.72	0.81	0.77	0.63	0.78	0.67
GMSD	0.94	0.96	0.89	0.94	0.92	**0.95**	**0.96**	0.90	**0.95**	0.92
PerceptNet	0.88	0.92	0.89	0.81	0.89	0.84	0.88	0.82	0.79	0.81
BioMultilayer	0.93	0.90	0.81	0.89	0.79	0.94	0.92	0.83	0.91	0.79

For each dataset, the highest-performing model is indicated in bold and the second highest with an underline.

Approach I with a Gaussian hypothesis improves drastically on the Euclidean null hypothesis, indicating that Gaussian connectivity plays a role; for the Euclidean versus approach I, Gaussian σ = 0.6 px (0.0310°), *p* < 0.001 (SceneIQ Online, two-tailed Fisher *r*-to-*z*; see Methods). However, the DOG hypothesis outperforms the Gaussian hypotheses; for approach I, Gaussian σ = 0.6 px (0.0310°) versus DOG, *p* < 0.001 (SceneIQ Online, two-tailed Fisher *r*-to-*z*). In predicting perceived distance judgments, supplementing Euclidean with approach I DOG scores, linear fit log(DMOS) ∼ log(Euclidean distance) + log(approach I DOG score) (adjusted *R*^2^ = 0.7166), is better than the Euclidean measure alone, with linear fit log(DMOS) ∼ log(Euclidean distance) (adjusted *R*^2^ = 0.1989; SceneIQ Online). More surprisingly, both approach I DOG and approach II tensors reliably outperform several performance-driven IQA algorithms (Table 1).

## Discussion

To the extent that the newly introduced perceptual displacement field does strain stimuli toward their relative perceived locations, we directly predict that this will explain effects in other perceptual domains such as color constancy, visual filling-in, category-specific connectivity, change blindness, and visual illusions. We are actively investigating some of these domains in our lab. For example, many disparate findings in visual crowding can unexpectedly be explained by a simple model that is almost identical to approach I, simply adding connectivity corresponding to increasing receptive field size that differs by degrees from fixation (Rodriguez & Granger, 2021).

It should be emphasized that the IQA tasks and corresponding datasets such as CSIQ and SceneIQ are unlikely to capture higher level aspects of perception. The approach presented here contains only simple connectivity profiles, speculated to be akin to early pathways, and cannot account for, for example, nonlinearities in categorical perception, top-down mechanisms in attention, or temporal dynamics in motion processing. Like the early visual stream (Dill & Fahle, 1998; Foster & Kahn, 1985; Nazir & O'Regan, 1990) but unlike higher level vision, such as face perception (Rolls, 2012; Wallis & Rolls, 1997), our approaches are not invariant to translation and scaling (Supplementary Materials).

Those higher level processes sit downstream from early vision. In the future, hierarchical and recurrent tensors may be important for capturing the above behavior. Approaches I and II may serve as valuable representations of the early visual pipeline, from which these more advanced models may draw. Human percepts are unlikely to derive from evenly weighted image regions, although weighting schemes have been previously proposed (e.g., Wang & Shang, 2006) and could eventually be applied here. Importantly, the brain may process distinct image regions differently depending on their content. Therefore, strain that is defined not as constant but as a function of the input may become an even more important extension.

These extensions will pose new challenges. The computational complexity of fitting these models is substantial, and differential geometric approaches will eventually suffer from being under-constrained, so methods must be devised to reduce the degrees of freedom. However, many popular approaches to gradient descent, evolution, sampling, and back-propagation take the Euclidean assumption—that optimization parameters can be evaluated in isolation. In the non-Euclidean case, motion along one dimension of the error manifold changes the shape of the manifold in all directions. One interesting path of future investigation will be to revise optimization algorithms to account for the interrelations among features being modeled. For example, under certain conditions, we believe that the optimization problem can be re-cast as a system of simple linear equations.

The underlying framework presented here also may be considered for a broader set of objectives. Models of perceived image quality are useful as error measures in an algorithmic search for superior image compression formulae (e.g., Romano, Isidoro, & Milanfar, 2017; Toderici et al., 2017), and reduce the need (in psychology, neuroscience, and software design) for high-volume human data collection (e.g., International Telecommunication Union, 1999; International Telecommunication Union, 2006). In machine learning, artificial networks are seldom designed with predetermined connectivity, although biologically informed connectivity in such networks has been advantageous (LeCun et al., 1989). In the future, a Jacobian can be cast as a predetermined weight matrix of an artificial network. The approaches of this paper may assist in further engineering solutions to analyses of multivariate data containing spatially or functionally related features. Examples include the decoding of signals from brain electrode data, functional magnetic resonance imaging, electrocorticography, computer vision, weather stations, or collaborative filtering.

We pose the IQA problem as one of perceptually deforming image space, using Cartesian coordinates of pixels (bitmaps) as the axes—the units of association. It is entirely possible that this projection from image to percept is more difficult than from other input feature spaces or coordinate axes. Discrete cosine style transforms have performed well in other IQA measures (e.g., Bradley, 1999; Lai & Kuo, 2000; Sampat, Wang, Gupta, Bovik, & Markey, 2009) but in our case were found to be inferior (Supplementary Figure S11). One explanation is that there are fewer neighborhood relations among features in those spaces, making them suboptimal for approaches based on feature–feature associations. Other coordinate axes (e.g., Narwaria & Lin, 2010; Sheikh & Bovik, 2006; Shnayderman, Gusev, & Eskicioglu, 2006; Zhang, Zhang, Mou, & Zhang, 2011) may consist of features whose relations more simply and consistently predict behavior. Ideally, it may be possible to find an embedding of the images in which feature relations strain to explain perception in an optimal or parsimonious way.

## Conclusions

Using well-known data from neural connectivity in the early visual pathway, we showed the ability to predict simple human similarity judgments (IQA fidelity), suggesting that much of the variance in these psychophysical judgments may be explained by surprisingly simple neural principles. Moreover, the predictions routinely rivaled or outperformed those of a standard approach in the field. It is also noteworthy that the formulations have been shown to also provide explanatory accounts of a broad range of psychophysical phenomena in the seemingly unrelated subfield of visual crowding (Rodriguez & Granger, 2021).

Neural representations are non-Euclidean; relations among neighboring features distort image geometry. Approach I explicitly replicates two non-Euclidean connectivity patterns within the visual system. Despite incorporating only quite simple neural principles, analyses indicate that approach I alone can equal or outperform industry-standard IQA measures at predicting human behavior.

Approach II further added the (still simple) refinement of data-driven regression. It should be emphasized that the regression was accomplished with a very small body of empirical measures (a regression fit to just 80 images generalizes nearly as well as one fit to 2080; see Supplementary Materials), as opposed to typical big-data methods. The regression found a set of pixel–pixel relations that perceptually strained images such that comparison between them was humanlike; the results are shown in Table 1 (see also Supplementary Materials).

The Jacobian matrices that result are directly interpretable in terms of connections among stimulus features. A given Jacobian directly represents hypotheses about connectivity in the early visual path, either from straightforward principled models (approach I) or derived from simply regressed behavioral data (approach II). The framework is therefore flexible, and many IQA approaches might be shown to be special cases when the restrictions of approaches I and II are lifted.

The more complex the relationship between predictions and empirical behavior, the more difficult it may be to unearth explanatory principles underlying predictive performance. It is hoped that the relatively straightforward methods forwarded here assist in the simplification of our understanding of similarity judgments.

We sought a formalism that quantifies connectivity in units of input–input relations (∂*u_i_*/∂*s_j_*), rather than input–output relations, *y* = $f(\overset{{}_{⇀}}{s})$, as is more typical in artificial neural network approaches. The findings suggest the potential of such formalisms to help us understand how patterns of individual associations yield the gestalt of an image percept, which is composed of many outputs working together rather than in isolation. Such a formalism is aligned with many insights neuroscientists have acquired about connectivity. Further characterization of the types of candidate hypotheses is in progress.

SceneIQ presents a new scale of dataset for IQA. It contains 69 times as many original (non-degraded) images as CSIQ, making analyses with higher order statistics or high-degree-of-freedom regressions reliable. Images in SceneIQ are evenly distributed among eight semantic categories, enabling future semantic analyses. The original images are well characterized in terms of image statistics and perception (e.g., Oliva & Torralba, 2001), and these characterizations are available to future study in IQA. The viewing conditions and subject pool are naturalistically variable, yet have been validated in a more controlled laboratory setting. Perhaps, the ecological validity of real, variable viewing conditions makes these ratings an even more reliable benchmark than scores collected under highly controlled conditions. We hope the depth of this dataset makes it a valuable benchmark for the field.

## Supplementary Material

Supplement 1
