# Supplementary material for: Visual stream connectivity predicts assessments of image quality
Source: J Vis. 2022 Oct 11;22(11):4. doi: 10.1167/jov.22.11.4 (PMC9580224; doi:10.1167/jov.22.11.4)
Supplement: Supplement 1 [file jovi-22-11-4_s001.pdf]

Supporting Information
Visual stream connectivity predicts assessments of image quality

Elijah F. W. Bowen, Antonio M. Rodriguez, Damian Sowinski, Richard Granger

Dartmouth

METHODS UNIQUE TO SUPPLEMENTAL FINDINGS

To account for nonlinearities in the Euclidean-DMOS and SSIM-DMOS relationships, we report in **Supplemental Table 3**, **Supplemental Fig S 3**, and **Supplemental Fig S 4** the fit of the logistic function in the equation below (in line with prior presentations (Chandler & Hemami, 2007) (Cheng, Huang, Zhu, Liu, & Cheng, 2010) (Ferzli & Karam, 2009) (Larson & Chandler, 2010); for similar logistic approaches, see also (Gao, Lu, Tao, & Li, 2009) (Kim, Han, & Park, 2010) (Moorthy & Bovik, 2010) (Sheikh, Bovik, & De Veciana, 2005) (Sheikh, Sabir, & Bovik, 2006) (Group, 2000) (Wang, Bovik, Sheikh, & Simoncelli, 2004) (Wang, Simoncelli, & Bovik, 2003)).

$$y = \frac{\beta_1 - \beta_2}{1 + exp\left(\frac{-x + \beta_3}{\beta_4}\right)} + \beta_2$$

Like others (Chandler & Hemami, 2007) (Sheikh, Bovik, & De Veciana, 2005), we used Nelder-Mead search (Lagarias, Reeds, Wright, & Wright, 1998) to fit this equation to the data.

To evaluate the amount of data approach $\mathbf{II}$ needs to form an accurate model, we introduce the "SceneIQ Online 80 Image" model, which was trained on a randomly selected subset of 80 images (10 per semantic scene category) from the SceneIQ Online dataset. The methods were identical to other approach $\mathbf{II}$ models (see paper).

This supplemental document contains results achieved on an additional "CSIQ Lab" dataset. These data were collected with the methodology of SceneIQ Lab, but with the stimuli from CSIQ Revised. CSIQ Lab is reported in this supplement for comparison.

FINDINGS

You will see five thumbnails: An "original" image (at the far left) and four "degraded" versions to the right of it. Your job is to rate each of the four images from 100 to 0 based on how different they are from the original.

100 means that the image is a perfect replica of the original

0 means that the image is completely degraded

Click on any two images to enlarge them. Left click (just regular click for mac) for the left zoom screen; right click (control-click for mac) for the right zoom screen.

You can click an enlarged image to deselect it.

Rate the images ONLY based on their enlarged versions, not the thumbnails.

Try comparing each image side by side with the original and with each other to make the most accurate judgments.

Once you're satisfied with your ratings, click "Accept".

Supplemental Fig S 1. Instructions to participants in the SceneIQ dataset.

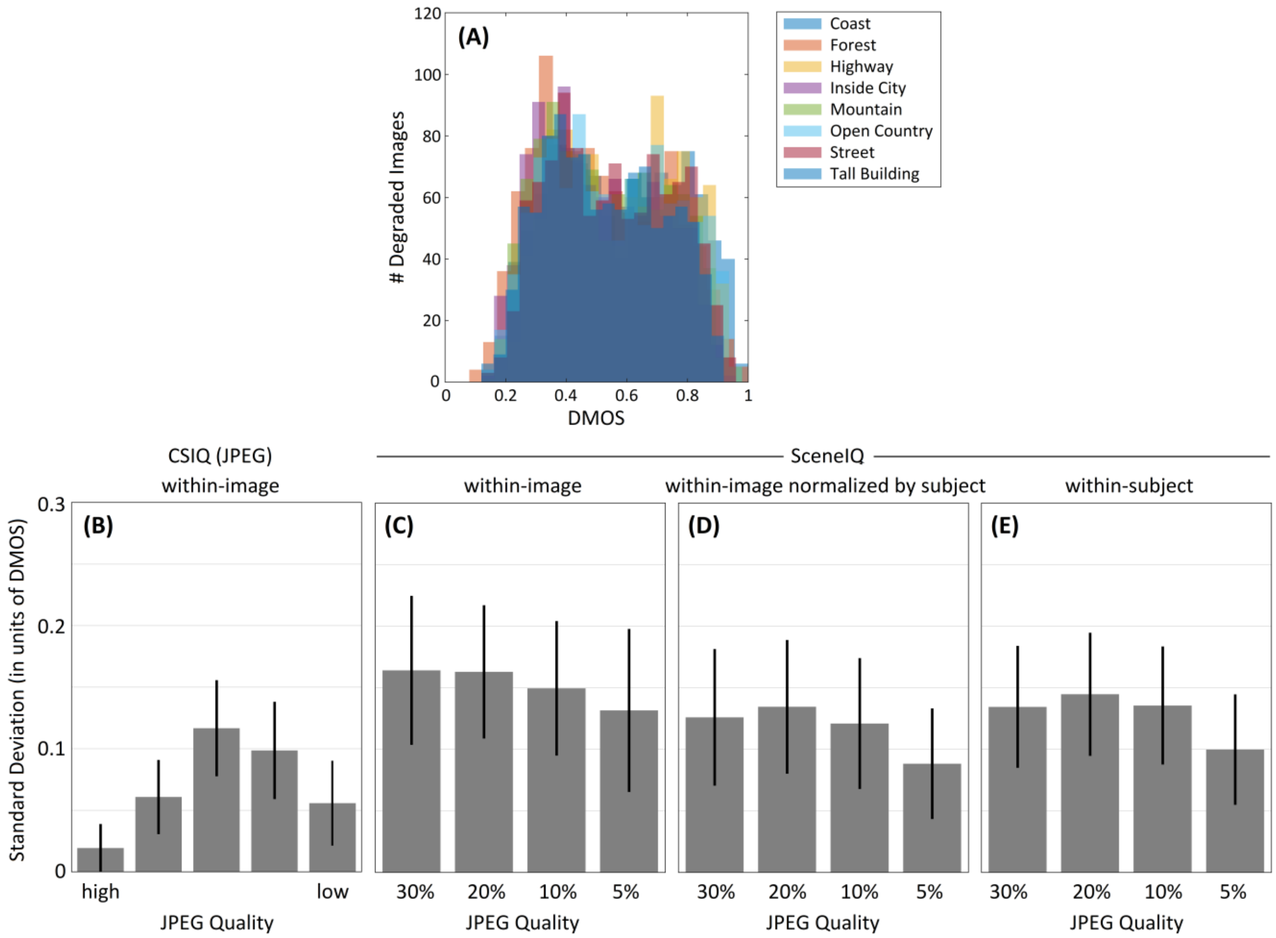

Supplemental Fig S 2. Summary statistics for the SceneIQ Online dataset. **(A)** Human DMOS scores fill out a large range of values from 0.1 to 1.0, regardless of semantic image category. These data may be slightly bimodal. **(B)** CSIQ (JPEG) dataset (for comparison). For each JPEG quality level, we plot the standard deviation of an image's DMOS ratings, meaned across images. Ranges indicated are standard deviation across images. **(C)** The same measurements for the SceneIQ dataset. Standard deviations of human scores can be an indication of dataset quality and reliability. The standard deviations trend higher for SceneIQ Online compared with CSIQ (JPEG), possibly due to variable viewing conditions. However, the higher number of images in the SceneIQ Online dataset increases the reliability of the mean. **(D)** Same as (C), but subjects were first mean-centered to remove the effect of the subject-wise mean on image-wise standard deviations. **(E)** SceneIQ Online dataset, standard deviation across images for each subject, meaned across subjects. Ranges indicated are standard deviation across subjects.

Supplemental Table 1

Spearman Correlation with Humans

		Approach I			Approach II				
		Gauss σ = 0.6	Gauss σ = 2	Center Surround	SceneIQ Online	SceneIQ Online 80 Img	SceneIQ Lab	Euclidean	SSIM
CSIQ (JPEG)		0.9376	**0.9582**	0.9578	0.9487	0.9484	0.9484	0.8883	0.9224
CSIQ Revised		0.7430	**0.8884**	0.8868	0.8760	0.8724	0.8715	0.5305	0.8104
CSIQ Lab		0.8028	0.9065	**0.9139**	0.9067	0.9042	0.9044	0.6137	0.8431
TID2013 (JPEG)		0.7981	0.5391	**0.9476**	0.9311	0.9268	0.9380	0.8782	0.9092
Toyama (JPEG)		0.4318	0.4554	0.5382	0.7605	0.7471	**0.7648**	0.4240	0.6505
SceneIQ Online	All	0.6760	0.6576	0.8295	**0.8314**	-	-	0.4738	0.7036
SceneIQ Online	C	0.6987	0.6042	**0.8597**	0.8574	-	-	0.5920	0.7550
SceneIQ Online	F	0.7723	0.7247	0.8356	0.8356	-	-	0.5238	**0.8417**
SceneIQ Online	H	0.7158	0.6472	**0.8616**	0.8605	-	-	0.6355	0.7108
SceneIQ Online	IC	0.8020	0.7347	0.8382	**0.8458**	-	-	0.7207	0.8207
SceneIQ Online	M	0.7283	0.6613	0.8380	**0.8461**	-	-	0.5585	0.7915
SceneIQ Online	OC	0.6911	0.5828	0.8462	**0.8470**	-	-	0.5107	0.7761
SceneIQ Online	S	0.8082	0.7443	0.8446	**0.8452**	-	-	0.7531	0.8362
SceneIQ Online	TB	0.7154	0.6015	0.8177	**0.8182**	-	-	0.6524	0.7751
SceneIQ Lab		0.7159	0.6570	0.8607	-	-	**0.8655**	0.5467	0.7567

Spearman correlation with humans (DMOS) on several datasets (rows). SceneIQ Online 80 Img is an approach **II** model fit to 80 out of the 2080 reference images (see **Supplemental Methods**). Euclidean distance between images and SSIM are included for comparison. Except SceneIQ, correlations were calculated on entire datasets. SceneIQ Online and SceneIQ Lab correlations represent the mean across two random folds of original (non-degraded) images (see **Methods**). Approach **II** SceneIQ Online was trained on each fold of SceneIQ Online and tested on the other fold (on other datasets, the reported correlation represents approach **II** fit to all SceneIQ Online, and tests the *generalization* of approach **II** trained on SceneIQ Online to a different dataset). For each dataset, the highest-performing model is marked in bold, and the second-highest with underline. All correlations were found to differ from zero with p << 0.001 via permutation test (after Bonferroni correction for 100 comparisons). C = coast, F = forest, H = highway, IC = inside city, M = mountain, OC = open country, S = street, TB = tall building. “Lab” = laboratory validation.

Supplemental Table 2

R^2 of Logistic Regression with Humans

		Approach I			Approach II				
		Gauss σ = 0.6	Gauss σ = 2	Center Surround	SceneIQ Online	SceneIQ Online 80 Img	SceneIQ Lab	Euclidean	SSIM
CSIQ (JPEG)		0.8571	**0.8860**	0.8690	0.5746	0.5779	0.5752	0.7524	0.7091
CSIQ Revised		0.5793	**0.8447**	0.8270	0.6487	0.6447	0.6446	0.3088	0.6061
CSIQ Lab		0.6564	**0.8584**	0.8554	0.6741	0.6716	0.6714	0.3894	0.6300
TID2013 (JPEG)		0.6326	0.2061	**0.9309**	0.8059	0.8059	0.8070	0.7397	0.7596
Toyama (JPEG)		0.1740	0.0987	0.2520	**0.4907**	0.4794	0.4900	0.1584	0.3639
SceneIQ Online	All	0.4053	0.2513	**0.7003**	0.5699	-	-	0.2086	0.4017
SceneIQ Online	C	0.3952	0.2490	**0.7147**	0.5695	-	-	0.2927	0.4558
SceneIQ Online	F	0.5741	0.4261	**0.7363**	0.6583	-	-	0.2751	0.6467
SceneIQ Online	H	0.3783	0.2095	**0.7346**	0.5627	-	-	0.3320	0.4145
SceneIQ Online	IC	0.6410	0.4385	**0.7449**	0.6561	-	-	0.5157	0.6516
SceneIQ Online	M	0.4580	0.2414	**0.7080**	0.6059	-	-	0.3089	0.5648
SceneIQ Online	OC	0.4062	0.2141	**0.7330**	0.6360	-	-	0.2539	0.5558
SceneIQ Online	S	0.6478	0.4217	**0.7356**	0.6457	-	-	0.5582	0.6796
SceneIQ Online	TB	0.4529	0.1992	**0.7003**	0.5948	-	-	0.4079	0.5641
SceneIQ Lab		0.4434	0.2312	**0.7523**	-	-	0.6291	0.2655	0.4783

R^2 of logistic regression with humans (DMOS). Same datasets (rows) and models (columns) as **Supplemental Table 1**. Some readers may find anecdotally that the relationship between models and humans is logistic in shape (see for example **Supplemental Fig S 3**).

Supplemental Table 3
Pearson Correlation with Humans

		Approach I			Approach II				
		Gauss σ = 0.6	Gauss σ = 2	Center Surround	SceneIQ Online	SceneIQ Online 80 Img	SceneIQ Lab	Euclidean	SSIM
CSIQ (JPEG)		0.9256	**0.9413**	0.9322	0.7580	0.7602	0.7584	0.8648	0.8410
CSIQ Revised		0.7590	**0.9191**	0.9094	0.8052	0.8026	0.8026	0.5488	0.7785
CSIQ Lab		0.8101	**0.9265**	0.9249	0.8210	0.8195	0.8194	0.6231	0.7937
TID2013 (JPEG)		0.7954	0.4538	0.9648	0.8977	0.8977	**0.8983**	0.8600	0.8715
Toyama (JPEG)		0.4163	0.3124	0.5020	**0.7002**	0.6919	0.6997	0.3979	0.6032
SceneIQ Online	All	0.6329	0.5011	**0.8335**	0.7559	-	-	0.4474	0.6327
SceneIQ Online	C	0.6285	0.4990	**0.8423**	0.7535	-	-	0.5405	0.6724
SceneIQ Online	F	0.7499	0.6465	**0.8559**	0.8119	-	-	0.5122	0.8042
SceneIQ Online	H	0.6139	0.4568	**0.8523**	0.7505	-	-	0.5750	0.6395
SceneIQ Online	IC	0.7944	0.6614	**0.8590**	0.8076	-	-	0.7092	0.8042
SceneIQ Online	M	0.6697	0.4887	**0.8382**	0.7778	-	-	0.5441	0.7480
SceneIQ Online	OC	0.6352	0.4608	**0.8535**	0.7986	-	-	0.4966	0.7441
SceneIQ Online	S	0.7980	0.6460	**0.8540**	0.8023	-	-	0.7361	0.8203
SceneIQ Online	TB	0.6708	0.4457	**0.8345**	0.7719	-	-	0.6342	0.7503
SceneIQ Lab		0.6637	0.4804	**0.8662**	-	-	0.7928	0.5106	0.6911

Pearson correlation with humans (DMOS) on linear axes. Same datasets (rows) and models (columns) as **Supplemental Table 1**.

Supplemental Table 4

Spearman Correlation with Humans

		Approach I			Approach II					
		Gauss σ = 0.6	Gauss σ = 2	Center Surround	SceneIQ Online	SceneIQ Online 80 Img	SceneIQ Lab	CSIQ Same Degradation	Euclidean	SSIM
CSIQ	(JPEG)	0.9351	0.9573	**0.9586**	0.9491	0.9484	0.9488	0.9447	0.8860	0.9211
	(jpeg2000)	0.9695	**0.9798**	0.9749	0.9689	0.9686	0.9684	0.9376	0.9435	0.9238
	(fnoise)	0.9343	0.9344	**0.9361**	0.9331	0.9332	0.9327	0.9322	0.9326	0.8938
	(blur)	**0.9708**	0.9537	0.9618	0.9683	0.9692	0.9678	0.9346	0.9384	0.9322
	(awgn)	**0.9449**	0.9055	0.9377	0.9370	0.9373	0.9351	0.9407	0.9413	0.9285

Pearson correlation with humans (DMOS) on log-log axes for the presented models (columns) on several datasets (rows). All values are calculated as the mean of two cross-validation folds of images. Same rows and columns as **Table 2** of the main paper.

Supplemental Table 5

Parson Correlation with Humans on TID2013

			AGN	AN	SCN	MN	HFN	IN	QN	GB	ID	JPG	JP2K	JXE	JP2K XE	NPN	BW	MIS	CCS	MGN	CN	LCNI	ICQ D	CA	SSR
Pearson Correlation on Log-log Axes	Approach I	Gaussian σ = 0.6 px (0.0310°)	0.76	0.71	0.81	0.43	0.86	0.79	0.64	0.81	0.87	0.79	0.88	0.76	0.77	0.70	0.08	0.77	0.10	0.80	0.73	0.81	0.72	0.91	0.84
		Gaussian σ = 2 px (0.1238°)	0.46	0.46	0.56	0.17	0.54	0.59	0.53	0.58	0.75	0.49	0.66	0.57	0.46	0.77	-0.11	0.75	0.10	0.53	0.39	0.59	0.70	0.86	0.69
		Center Surround (DOG)	0.93	0.91	0.92	0.70	0.95	0.87	0.85	0.86	0.95	0.96	0.94	0.79	0.80	0.78	0.31	0.78	0.04	0.89	0.93	0.95	0.78	0.92	0.94
	Appr. II	SceneIQ Online	0.95	0.90	0.94	0.62	0.94	0.89	0.86	0.92	0.97	0.95	0.97	0.77	0.88	0.76	0.36	0.67	-0.08	0.91	0.92	0.96	0.81	0.93	0.98
		SceneIQ Lab	0.95	0.90	0.94	0.65	0.95	0.89	0.86	0.93	0.97	0.95	0.97	0.78	0.88	0.75	0.36	0.67	-0.08	0.91	0.92	0.96	0.82	0.93	0.98
		Euclidean (MSE)	0.90	0.87	0.88	0.76	0.94	0.85	0.74	0.82	0.90	0.86	0.91	0.78	0.82	0.66	0.12	0.78	0.09	0.87	0.82	0.90	0.77	0.94	0.90
		SSIM	0.77	0.75	0.75	0.79	0.80	0.69	0.74	0.81	0.84	0.89	0.84	0.80	0.75	0.76	0.59	0.59	-0.45	0.70	0.74	0.88	0.67	0.91	0.86
		MSSIM	0.77	0.75	0.73	0.78	0.80	0.64	0.74	0.78	0.86	0.89	0.85	0.80	0.74	0.74	0.44	0.66	-0.44	0.71	0.71	0.85	0.68	0.82	0.84
		IWSSIM	0.74	0.73	0.70	0.82	0.79	0.60	0.72	0.78	0.85	0.86	0.81	0.81	0.74	0.79	0.34	0.65	-0.47	0.69	-	0.81	-	0.75	0.78
		VSNR	0.82	0.77	0.76	0.67	0.85	0.62	0.77	0.85	0.88	0.93	0.89	0.76	0.70	0.67	0.14	0.55	-0.26	0.75	0.87	0.88	-	0.88	0.83
		VIF	0.88	0.87	0.85	0.87	0.92	0.82	0.81	0.87	0.91	0.94	0.91	0.82	0.82	0.80	0.48	0.45	-0.38	0.84	0.87	0.88	0.83	0.88	0.89
		VIFP	0.82	0.80	0.82	0.86	0.88	0.79	0.78	0.91	0.90	0.92	0.91	0.82	0.83	0.80	0.49	0.52	-0.39	0.76	0.83	0.87	0.77	0.92	0.90
		IFC	0.64	0.56	0.61	0.74	0.74	0.59	0.60	0.79	0.79	0.82	0.86	0.70	0.72	0.25	0.04	0.45	-0.43	0.60	0.78	0.77	0.58	0.82	0.84
		GMSD	0.91	0.88	0.92	0.60	0.90	0.72	0.88	0.87	0.96	0.96	0.97	0.86	0.88	0.82	0.61	0.67	-0.20	0.85	0.95	0.96	0.86	0.96	0.98
		PerceptNet	0.74	0.60	0.70	0.67	0.82	0.74	0.38	0.66	0.77	0.67	0.82	0.62	0.63	0.76	0.37	0.50	0.25	0.72	0.35	0.62	0.71	0.80	0.83
		BioMultilayer	0.81	0.77	0.82	0.48	0.87	0.79	0.78	0.88	0.93	0.93	0.94	0.83	0.85	0.79	0.49	0.68	-0.27	0.82	0.87	0.91	0.75	0.95	0.93
Pearson Correlation on Linear Axes	Approach I	Gaussian σ = 0.6 px (0.0310°)	0.75	0.71	0.81	0.43	0.87	0.80	0.66	0.82	0.90	0.80	0.89	0.77	0.78	0.70	0.09	0.78	0.10	0.80	0.73	0.81	0.71	0.92	0.85
		Gaussian σ = 2 px (0.1238°)	0.42	0.44	0.51	0.16	0.51	0.55	0.55	0.58	0.76	0.46	0.64	0.53	0.40	0.76	-0.12	0.77	0.10	0.49	0.35	0.57	0.66	0.85	0.68
		Center Surround (DOG)	0.94	0.92	0.93	0.70	0.96	0.87	0.85	0.84	0.96	0.97	0.95	0.79	0.79	0.76	0.33	0.80	0.05	0.89	0.93	0.96	0.75	0.93	0.94
	Appr. II	SceneIQ Online	0.89	0.88	0.88	0.53	0.87	0.81	0.74	0.70	0.85	0.90	0.89	0.67	0.73	0.65	0.39	0.81	-0.04	0.85	0.81	0.90	0.61	0.87	0.90
		SceneIQ Lab	0.90	0.89	0.88	0.57	0.88	0.81	0.74	0.71	0.84	0.90	0.89	0.67	0.73	0.65	0.39	0.80	-0.04	0.85	0.82	0.90	0.61	0.88	0.90
		Euclidean (MSE)	0.91	0.87	0.89	0.76	0.95	0.86	0.76	0.84	0.92	0.86	0.91	0.78	0.83	0.66	0.13	0.78	0.09	0.87	0.82	0.90	0.77	0.95	0.91
		SSIM	0.75	0.75	0.73	0.79	0.74	0.66	0.74	0.80	0.83	0.88	0.82	0.78	0.72	0.75	0.60	0.60	-0.45	0.69	0.64	0.88	0.53	0.91	0.84
		MSSIM	0.78	0.76	0.73	0.79	0.81	0.64	0.75	0.82	0.89	0.91	0.87	0.82	0.74	0.73	0.45	0.68	-0.44	0.71	0.71	0.87	0.68	0.84	0.86
		IWSSIM	0.75	0.73	0.69	0.83	0.80	0.60	0.72	0.82	0.88	0.87	0.81	0.82	0.74	0.79	0.34	0.67	-0.47	0.67	-	0.82	-	0.74	0.77
		VSNR	0.82	0.78	0.77	0.68	0.86	0.62	0.78	0.86	0.90	0.93	0.91	0.78	0.71	0.67	0.12	0.54	-0.24	0.76	0.88	0.89	-	0.89	0.84
		VIF	0.87	0.87	0.82	0.88	0.90	0.80	0.80	0.82	0.86	0.88	0.68	0.72	0.79	0.80	0.49	0.48	-0.38	0.82	0.66	0.75	0.79	0.58	0.63
		VIFP	0.80	0.79	0.77	0.86	0.86	0.76	0.77	0.87	0.87	0.88	0.83	0.74	0.78	0.80	0.51	0.55	-0.39	0.74	0.71	0.81	0.73	0.80	0.79
		IFC	0.63	0.56	0.61	0.75	0.72	0.58	0.59	0.79	0.78	0.77	0.77	0.63	0.70	0.24	0.03	0.45	-0.43	0.59	0.65	0.72	0.58	0.69	0.74
		GMSD	0.91	0.89	0.92	0.60	0.92	0.72	0.89	0.89	0.97	0.97	0.98	0.88	0.89	0.81	0.63	0.69	-0.19	0.86	0.95	0.97	0.87	0.97	0.98
		PerceptNet	0.71	0.58	0.64	0.69	0.84	0.67	0.51	0.63	0.77	0.62	0.83	0.65	0.58	0.73	0.40	0.56	0.19	0.71	0.24	0.56	0.63	0.81	0.81
		BioMultilayer	0.81	0.77	0.83	0.48	0.88	0.80	0.80	0.91	0.95	0.94	0.96	0.85	0.86	0.78	0.51	0.70	-0.27	0.83	0.89	0.92	0.76	0.97	0.95

Pearson correlation with humans (DMOS) on log-log and on linear axes for the presented models (rows) on several TID2013 sub-datasets (columns). AGN = additive Gaussian noise, AN = additive noise in color components is more intensive than additive noise in the luminance component, SCN = spatially correlated noise, MN = masked noise, HFN = high frequency noise, IN = impulse noise, QN = quantization noise, GB = Gaussian blur, ID = image denoising, JPG = JPEG compression, JP2K = JPEG2000 compression, JXE = JPEG transmission errors, JP2KXE = JPEG2000 transmission errors, NPN = non-eccentricity pattern noise, BW = local block-wise distortions of different intensity, MIS = mean intensity shift, CCS = change of color saturation MGN = multiplicative Gaussian noise, CN = comfort noise, LCNI = lossy compression of noisy images,

ICQD = Image color quantization with dither, CA = chromatic aberrations, SSR = sparse sampling and reconstruction. The "contrast change" subset was omitted due to issue with the human ratings.

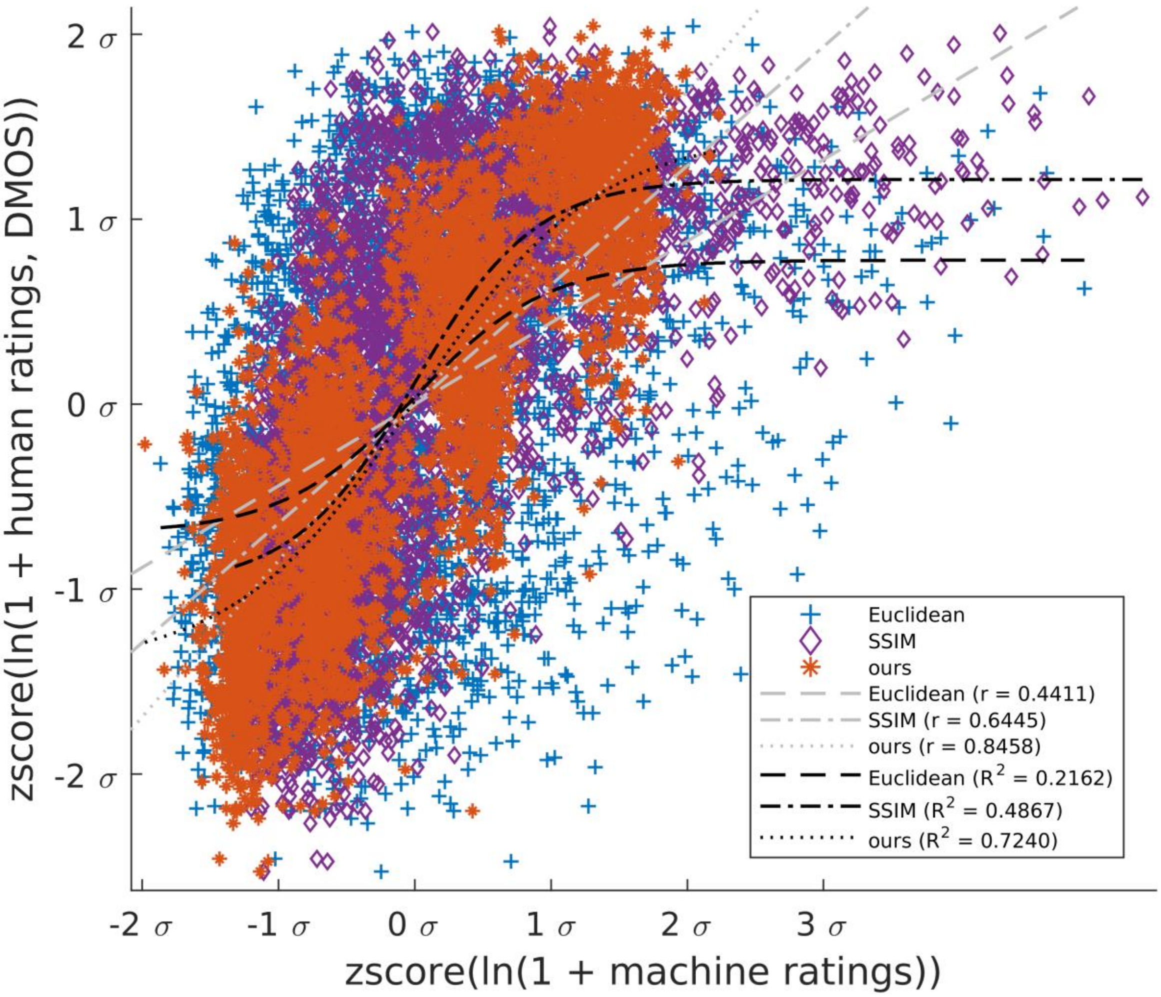

Supplemental Fig S 3. Correlation of Euclidean, SSIM, and approach **II** with DMOS (log-log axes). Half of SceneIQ Online (cross-validation fold 1). Comparable to **Fig 3** of the main paper. Machine ratings are on the X-axis, while human DMOS ratings are on the Y axis. Each set of machine ratings was z-scored separately so that they can be more usefully superimposed. We plot lines and logistic correlations of best fit: Euclidean (R^2 = 0.2162) y = 0.0794 / (1 + exp((-x+0.0000)/0.4879)) - 0.6999, SSIM (R^2 = 0.4867) y = 0.2322 / (1 + exp((-x+0.0000)/0.4434)) - 0.9837, approach **II** (R^2 = 0.7240) y = 0.0428 / (1 + exp((-x-0.0000)/0.6628)) - 1.4256.

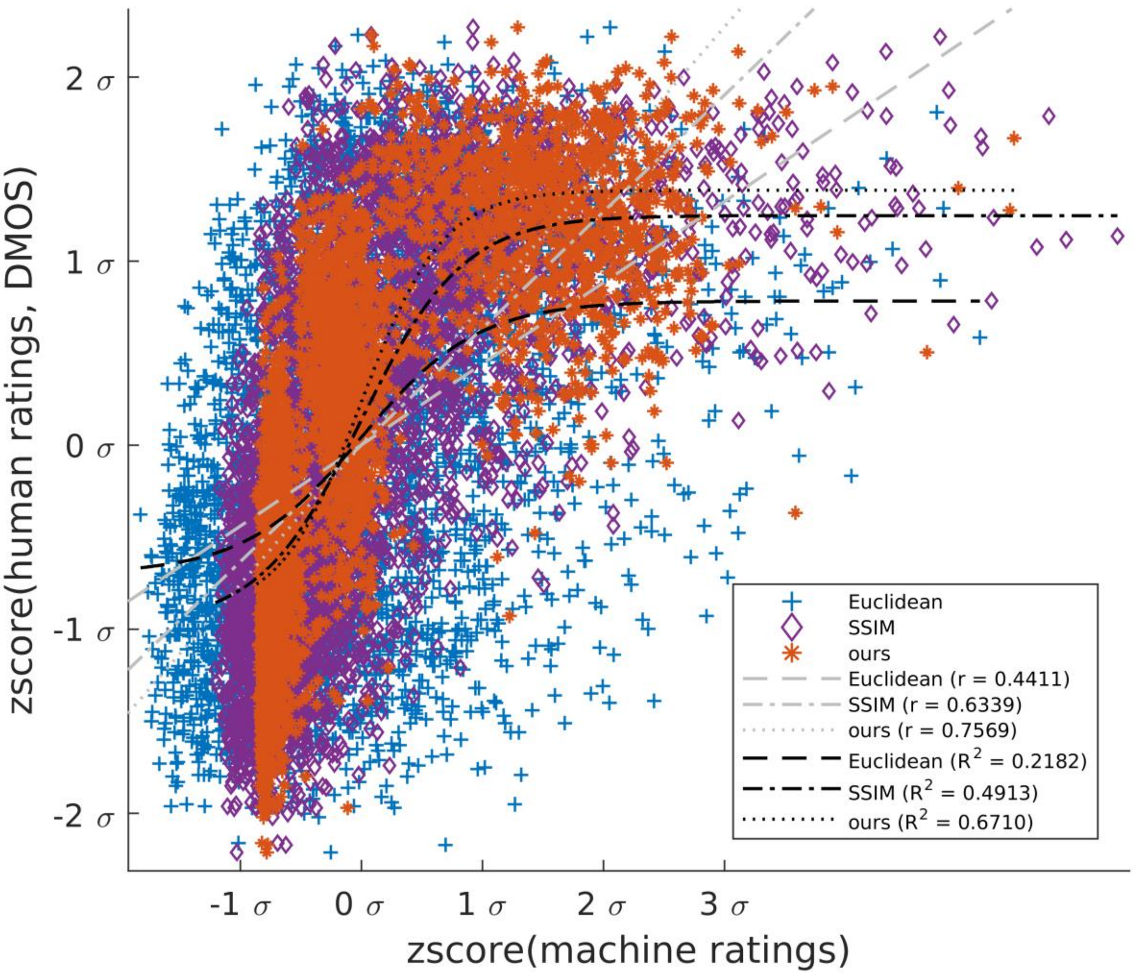

Supplemental Fig S 4. Correlation of Euclidean, SSIM, and approach **II** with DMOS (linear axes). Half of SceneIQ Online (cross-validation fold 1). Comparable to **Fig 3** of the main paper. Machine ratings are on the X-axis, while human DMOS ratings are on the Y axis. Each set of machine ratings was z-scored separately so that they can be more usefully superimposed. We plot lines and logistic correlations of best fit: Euclidean (R^2 = 0.2182) y = 0.0858 / (1 + exp((-x-0.0000)/0.4802)) - 0.6977, SSIM (R^2 = 0.4913) y = 0.2721 / (1 + exp((-x+0.0000)/0.4218)) - 0.9758, approach **II** (R^2 = 0.6710) y = 0.4669 / (1 + exp((-x+0.0000)/0.3363)) - 0.9184.

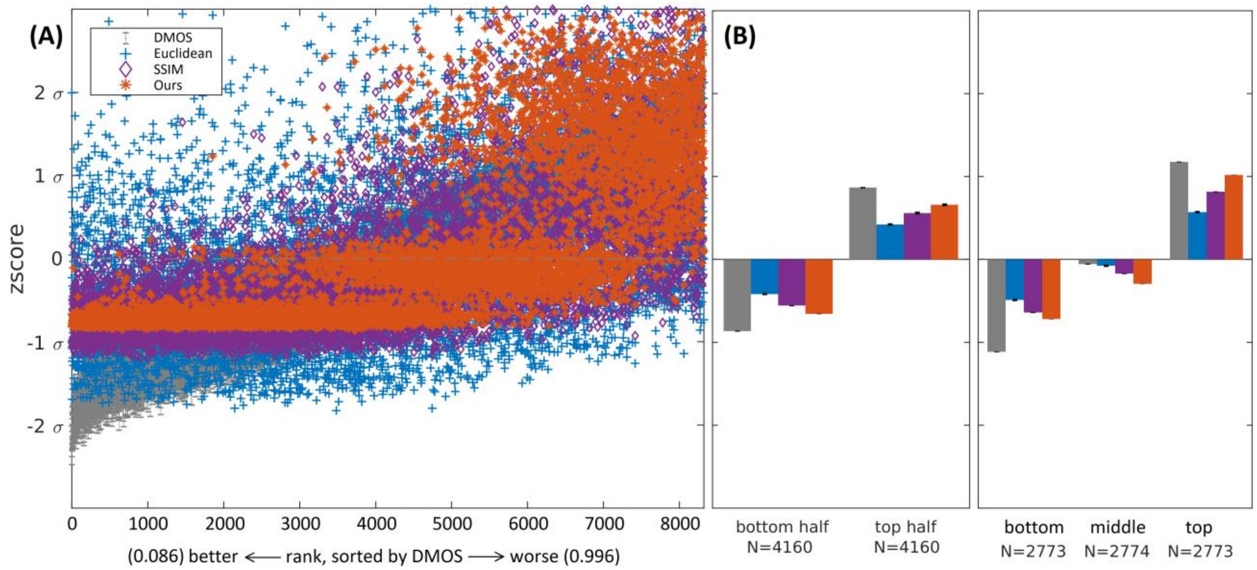

Supplemental Fig S 5. Correlation of Euclidean, SSIM, and approach **II** with DMOS within and between parts of the DMOS ordinality. **(A)** The X-axis is an ordinal scale, containing individual stimuli - the 8,320 degraded images of SceneIQ Online, combined across categories and JPEG degradation levels, and ranked by ascending DMOS scores. The Y-axis contains (z scores of) both human empirical measures (DMOS (o)) and estimated measures (Euclidean distance (+), SSIM (◊), and approach **II** trained on SceneIQ Online (*)). It can be seen that Euclidean distance (+) performs poorly. SSIM (◊) performs relatively well in the low-DMOS domain, but less well in the middle and high domains. Approach **II** (*) performs most closely to actual DMOS scores in all three ranges. **(B)** Bars (left to right: DMOS in gray, Euclidean in blue, SSIM in purple, approach **II** in orange) represent the mean values (± standard error) based on 2-way and 3-way splits of the X-axis in (A).

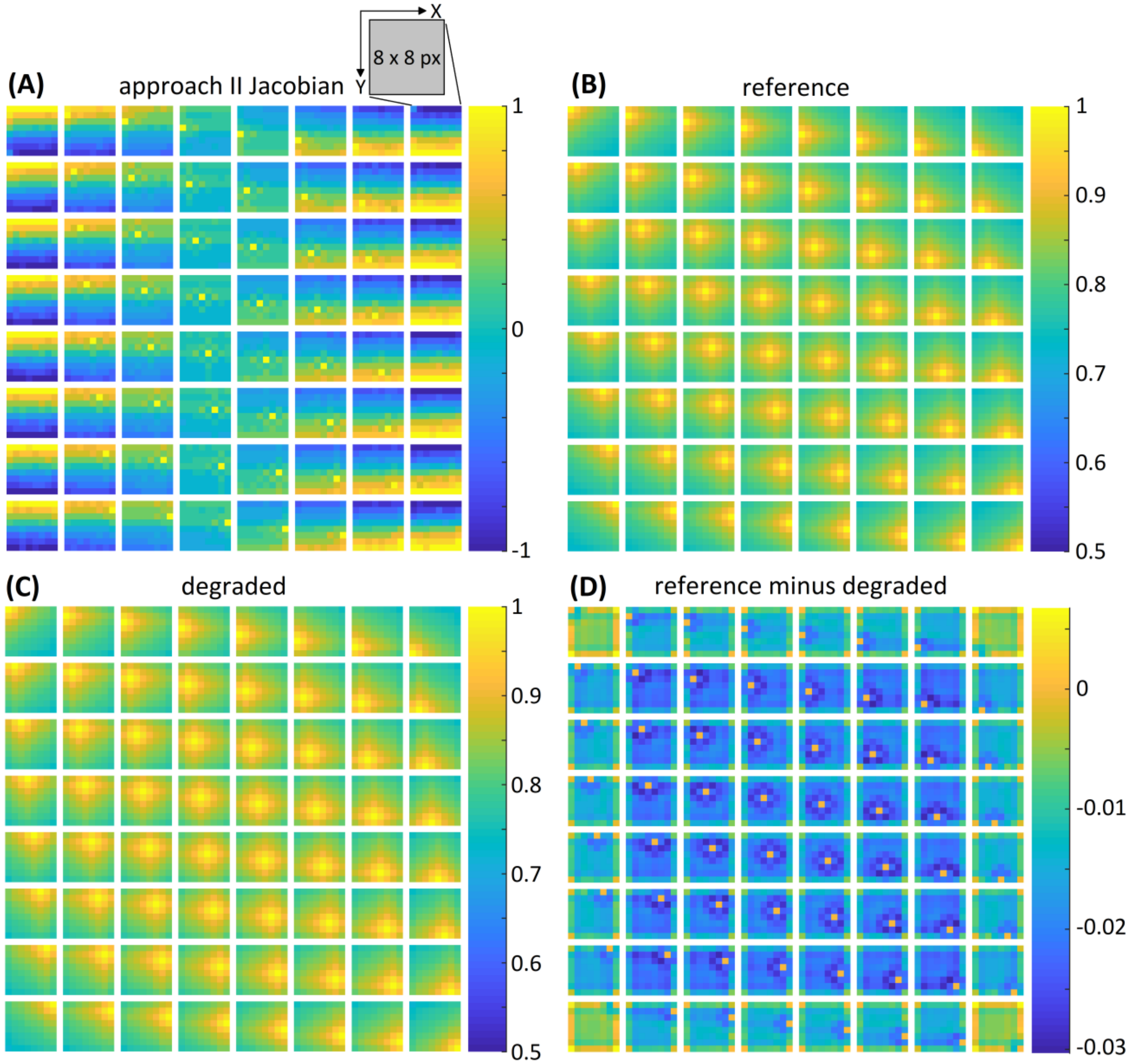

Supplemental Fig S 6. The degree to which each pixel associates with neighboring pixels. Each 8 x 8 block is a single column from the previous figure, converted from a vector back into (x,y) pixel coordinates. **(A)** Here, we rearranged the Jacobian from approach **II** (optimized for all images of SceneIQ Online) to show the degree to which each pixel is perceptually associated with neighboring pixels. The pixel along the primary diagonal of the Jacobian is in bright yellow. Pixels still correlate most strongly with their neighbors. Interestingly, pixels seem to inversely correlate with pixels far away on the Y axis, but this effect is less strong for the X-axis. This could be due to a human perceptual phenomenon, or could be an artifact caused by the optimization code finding one local minimum among several. For comparison, pixel-pixel intensity Pearson correlation was measured for each block in **(B)** reference images, and **(C)** degraded images. **(D)** Pixel-pixel correlation in reference images minus pixel-pixel correlation in degraded images. Correlation is measured relative to a single pixel, displayed in yellow (because it maximally correlates with itself).

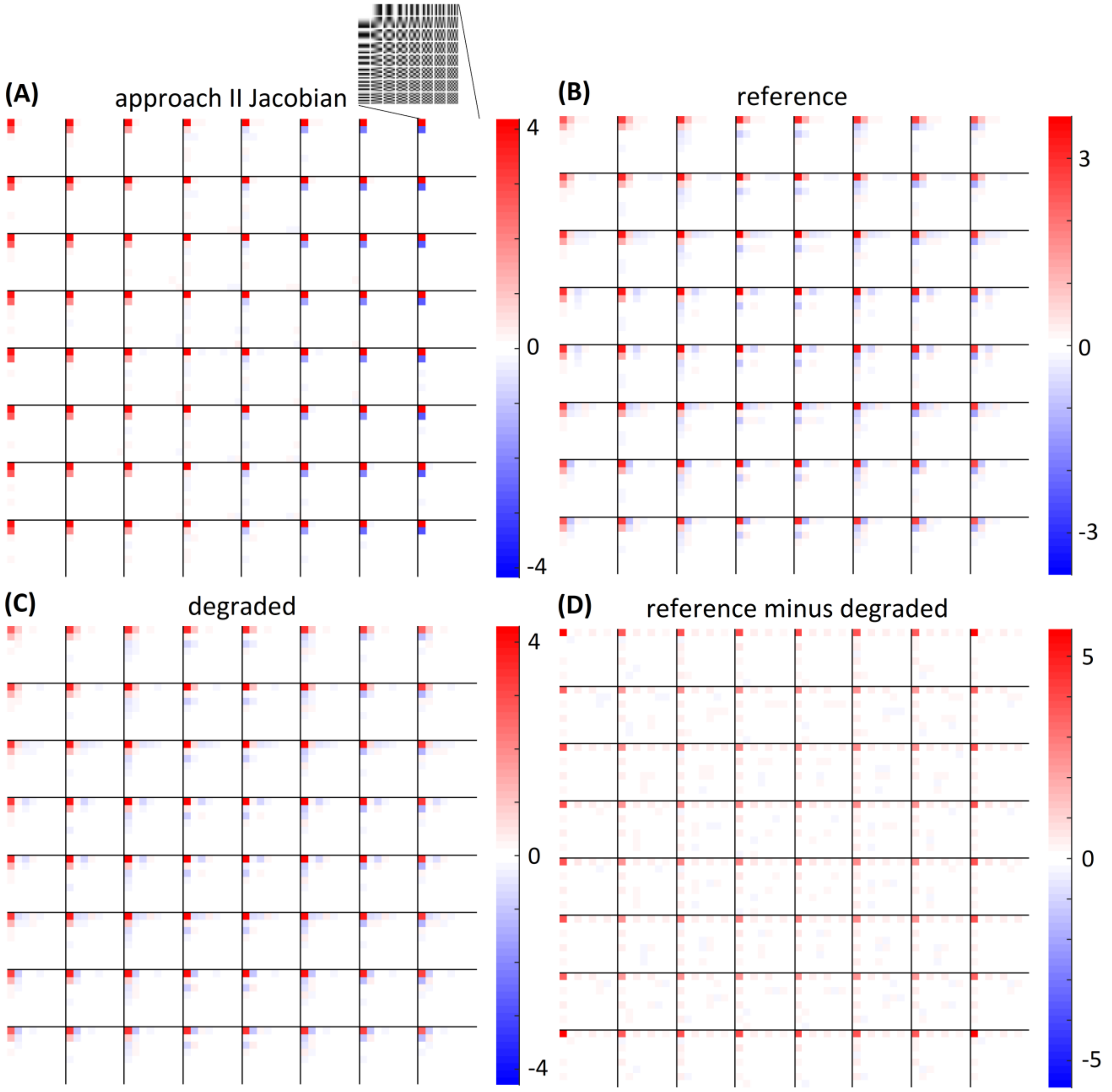

Supplemental Fig S 7. DCT transform of an approach **II** Jacobian and of related image statistics. Since JPEG compression operates on features of the 2D pixel DCT transform, we can inspect the DCT of pairwise correlation matrices and Jacobians. Each 8 x 8 px image patch in **Supplemental Fig S 6** was separately DCT transformed. **(A)** DCT of the approach **II** Jacobian after training on all of SceneIQ Online. Also, DCT of pairwise correlation between pixels in all of the SceneIQ Online **(B)** reference images, **(C)** degraded images, and **(D)** reference minus degraded images.

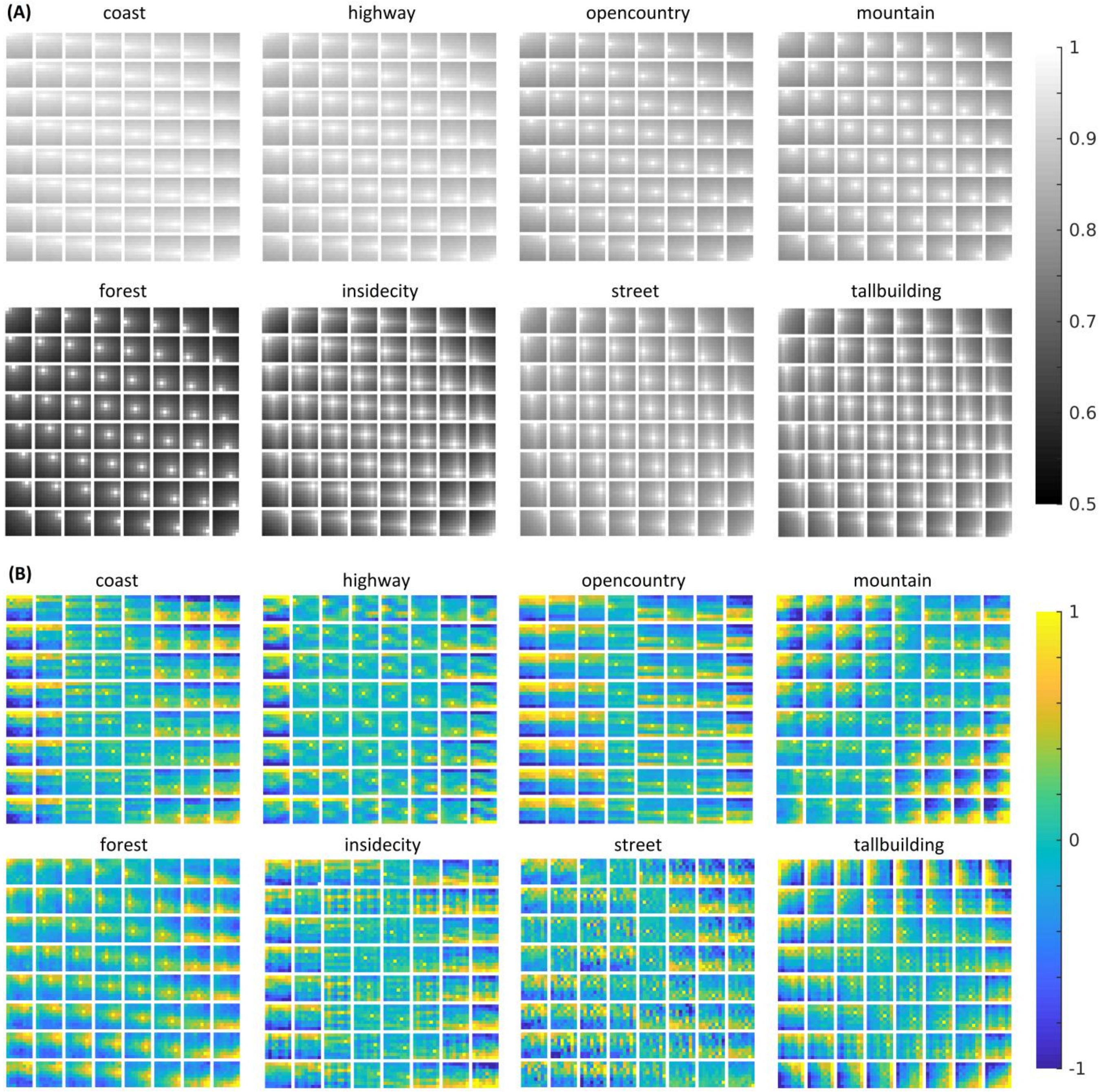

Supplemental Fig S 8. Pixel-pixel associations for each semantic category in SceneIQ Online (all images). Again, each 8 x 8 subimage renders how much a pixel (bright yellow/white) is associated with its neighbors. **(A)** Pixel-pixel intensity Pearson correlation in reference the images of each semantic category. Coastal scenes have greater correlation in the horizontal direction, and high correlation overall, indicating smooth surfaces divided by horizontal lines (e.g. horizon, waves). By contrast, high-spatial-frequency information dominates forest scenes. Although each individual pixel in a forest scene likely has one of several categories of spatial correlation (e.g. verticality in tree trunk pixels), there is no consistent high-spatial-distance pattern across all pixels. This indicates one weakness of approaches, such as the correlation in (A), that group pixels regardless of their context. In artificial scenes, vertical and horizontal lines dominate. **(B)** Approach **II** Jacobians, fit to each semantic category of scene in SceneIQ Online individually. The Jacobians tend to match the natural image statistics presented in (A). However, they seem to commit to spatial patterns that are likely dominant, but averaged out in the overall correlations. For example, the horizontal bias in the opencountry-fit Jacobian is far stronger than in the pixel-pixel Pearson correlations of opencountry in (A). The insidecity-fit Jacobian identifies not just vertical and horizontal lines, but grids that might represent building windows. Note also that the fit Jacobians tend to show an edge effect - because they were fit to 8x8 image patches, pixels in the corners were unable to fully form pixel-pixel associations that matched spatially broad patterns such as horizontal lines.

Supplemental Table 5

Entire SceneIQ Online Dataset Vs A Subset of 80 Images

	**CSIQ (JPEG)**	**CSIQ Revised**	**TID2013 (JPEG)**	**Toyama (JPEG)**
Approach II (trained on SceneIQ Online)	0.9487	0.8760	0.9311	0.7605
Approach II (trained on SceneIQ Online subset of 80)	0.9484	0.8724	0.9268	0.7471
p (difference)	0.9817	0.9062	0.8080	0.8439
Pearson correlation with one another	0.9998	0.9999	0.9997	0.9965

Spearman correlation with DMOS for approach **II** trained on the entire SceneIQ Online dataset and on a subset of 80 images. We also report the probability of difference between these two models, calculated with Fisher's r-to-z. These p values are not corrected for multiple (4) comparisons. Finally, we report the Pearson correlation between ratings produced by the two models (all correlations differ from chance by $p \ll 0.001$ after Bonferroni correction for 4 comparisons).

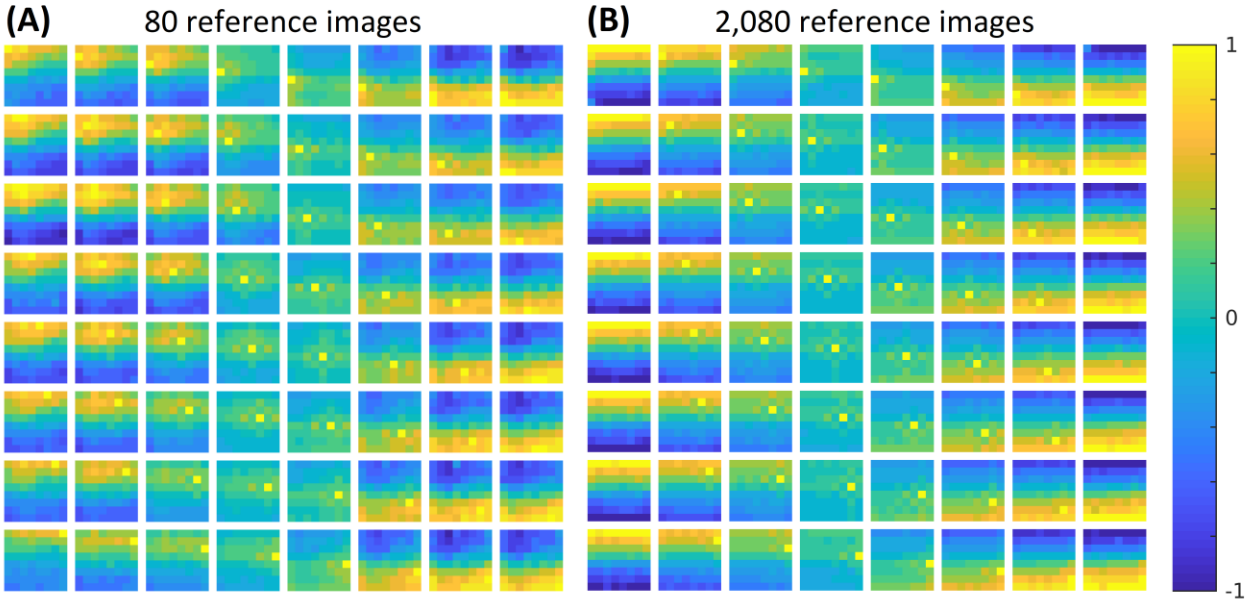

Supplemental Fig S 9. Comparison of approach **II** trained on all SceneIQ Online and on a small subset of 80 reference images. **(A)** Jacobian fit to model a small subset (80 reference images; 10 per semantic category) of SceneIQ Online. **(B)** Jacobian fit to model the entire SceneIQ Online dataset. Color bar applies to both. We find little difference between the two Jacobians. It is surprising that so few images are needed. This may be due to high image consistency within scene categories, consistent DMOS ratings, and a smooth regression error surface.

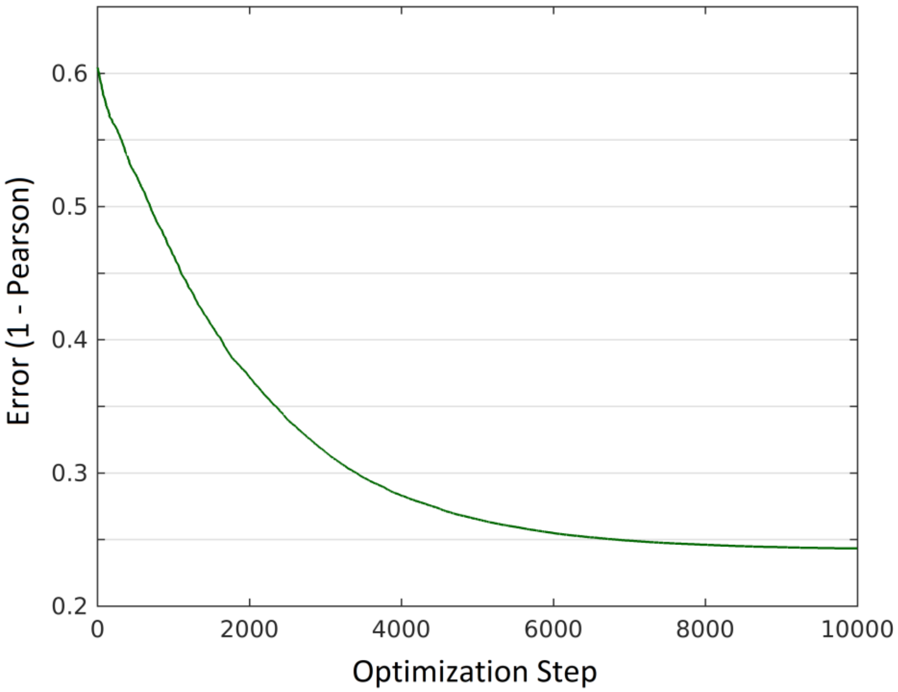

Supplemental Fig S 10. Error of approach **II** trained on all of SceneIQ Online, as training iterated. Step 1 is the error evaluated after one step from the Euclidean Jacobian. The rightmost step is the final Jacobian evaluated in this paper. On the Y axis is error, defined as 1 - Pearson correlation with human DMOS scores.

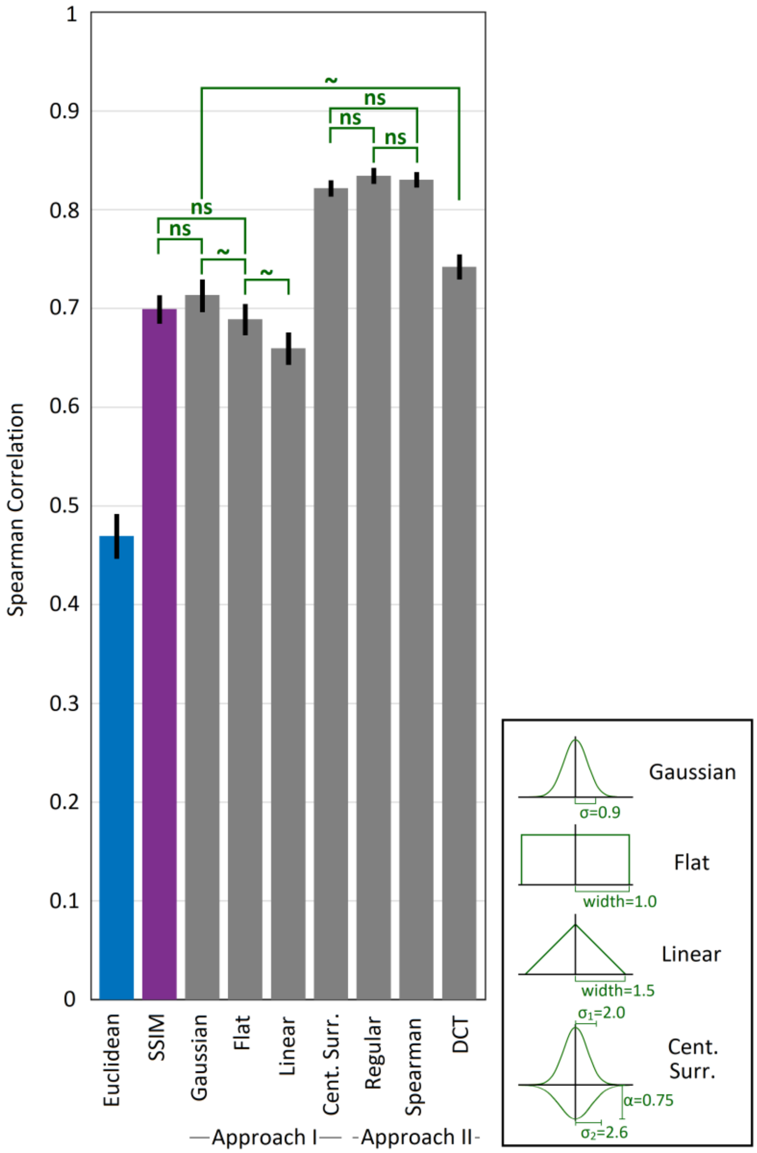

Supplemental Fig S 11. Performance of alternative IQA models on the first fold of 2-fold SceneIQ Online. Euclidean, SSIM, and various approach **I** and approach **II** alternatives are compared via Spearman correlation with DMOS. Bars are 95% bootstrapped confidence intervals (10,000 samples). All pairwise comparisons are significant (after conservative Bonferroni correction for 36 pairwise comparisons) except for those marked "non-significant" (**ns**) or "significant before Bonferroni correction" (**~**). Approach **I** alternatives were each independently parameterized to maximize Pearson correlation with SceneIQ Online training data. Approach **I** with Gaussian connectivity (as presented in main text) is not significantly worse than approach I with linear or flat connectivity functions. We also compare a center-surround topology computed as the difference between a center Gaussian (width σ_1), a surround Gaussian (width σ_2), and a scaling multiplier upon the surround Gaussian of α (as presented in main text). This model significantly outperforms other approach I connectivity profiles, and warrants further exploration. Approach **II** "Regular" is the model presented in main text. Approach **II** "Spearman" was regressed to fit an error function of 1 – Spearman correlation (instead of 1 – Pearson). Approach **II** "DCT" was fit just like "Regular," but was computed on the DCT transform of the images. In this case the features our Jacobian associates are not pixel intensities but DCT component weightings.

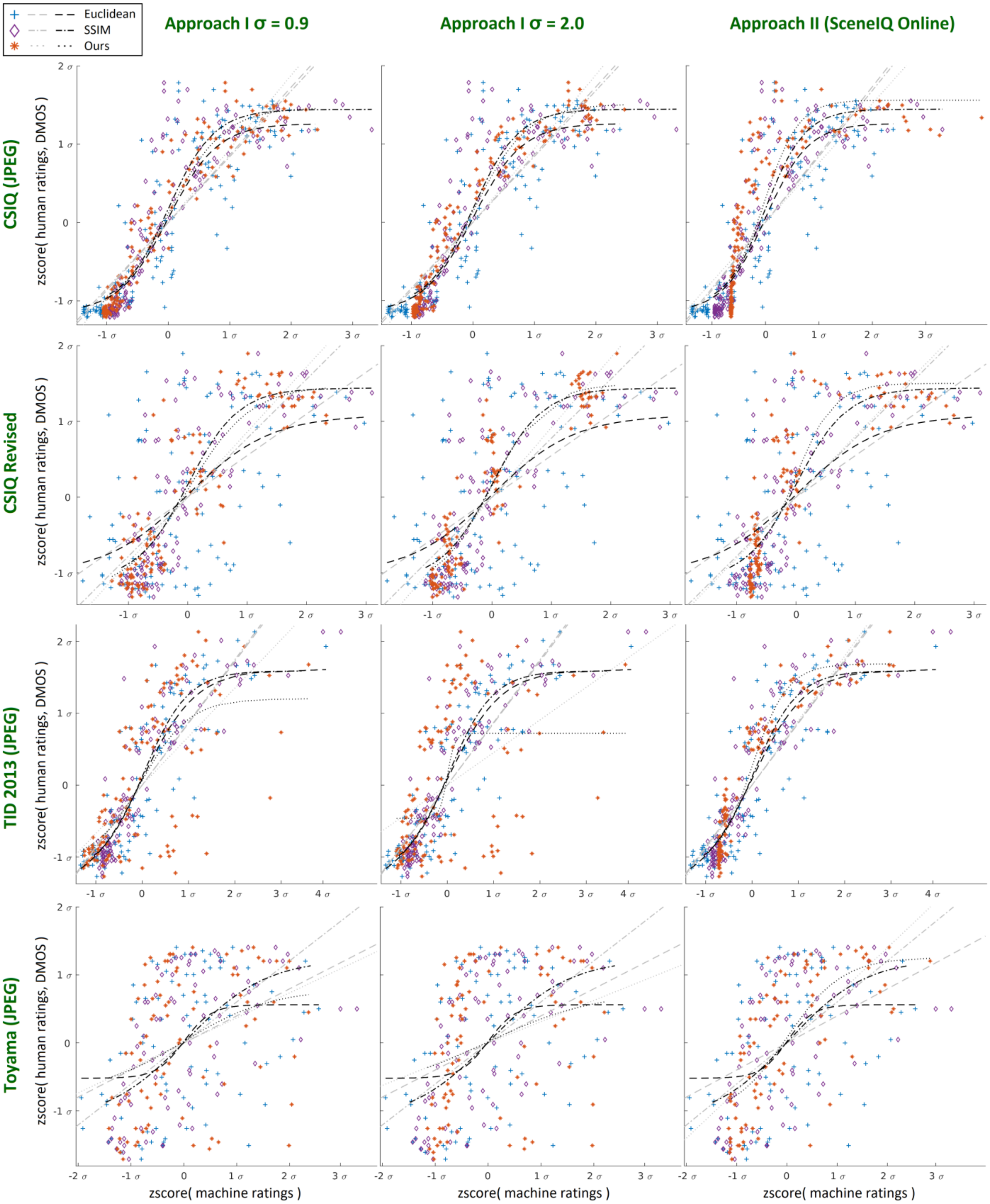

Supplemental Fig S 12. Approaches **I** and **II** fit to industry datasets. Correlation of Euclidean (blue), SSIM (purple), and our models (orange) with DMOS. Corresponding statistics are available in **Supplemental Table 3**. **Column 1** presents approach **I** σ = 0.9 pixels. **Column 2** presents approach **I** σ = 2.0 pixels. **Column 3** presents approach **II** trained on the full SceneIQ Online dataset. **Row 1**: CSIQ (JPEG); **Row 2**: CSIQ Revised; **Row 3**: TID 2013 (JPEG); **Row 4**: Toyama (JPEG). Machine ratings are on the X-axis, while human DMOS ratings are on the Y axis. Each set of machine ratings was z-scored separately so that they can be more usefully superimposed.

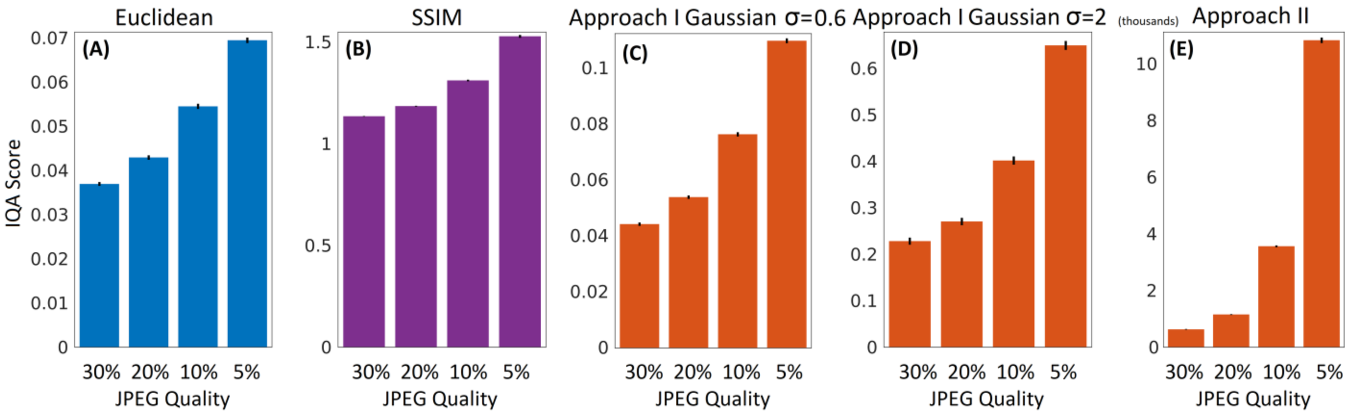

Supplemental Fig S 13. IQA scores by JPEG quality level. SceneIQ Online dataset, first half of images from the 2-fold analysis. Similar to **Fig 4D**, but for IQA measures rather than DMOS. **(A)** Euclidean distance, **(B)** SSIM, **(C)** Approach **I** Gaussian $\sigma = 0.6$ pixels, **(D)** Approach **I** Gaussian $\sigma = 2.0$ pixels, and **(E)** Approach **II** (trained on SceneIQ Online 2-fold). Bars are standard error across images.

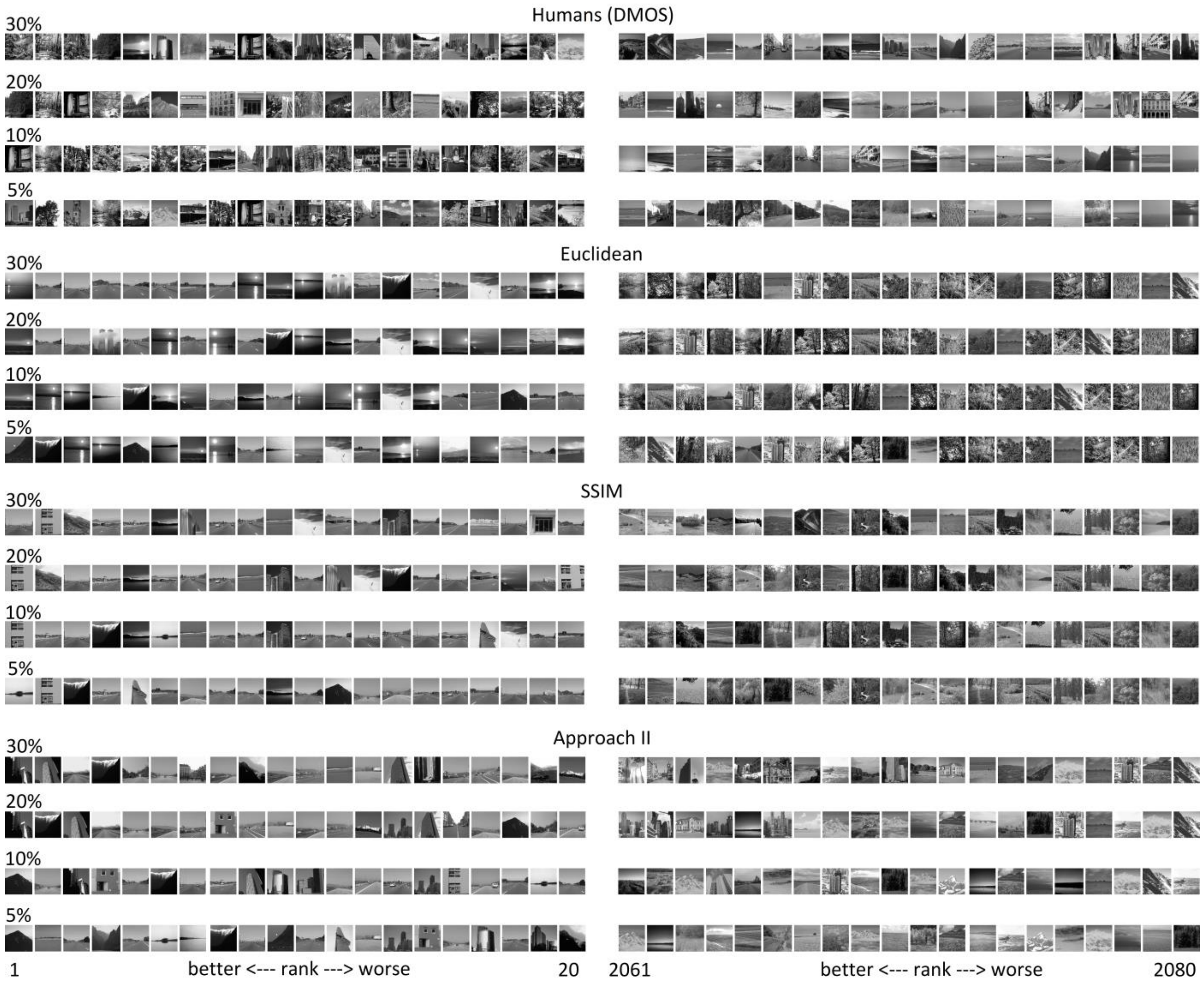

Supplemental Fig S 14. 20 lowest fidelity images and 20 highest fidelity images, as scored by humans (DMOS) and various models. SceneIQ Online dataset. Sorted for the set of all images (combined across categories). Approach **II** was fit to entire SceneIQ Online dataset.

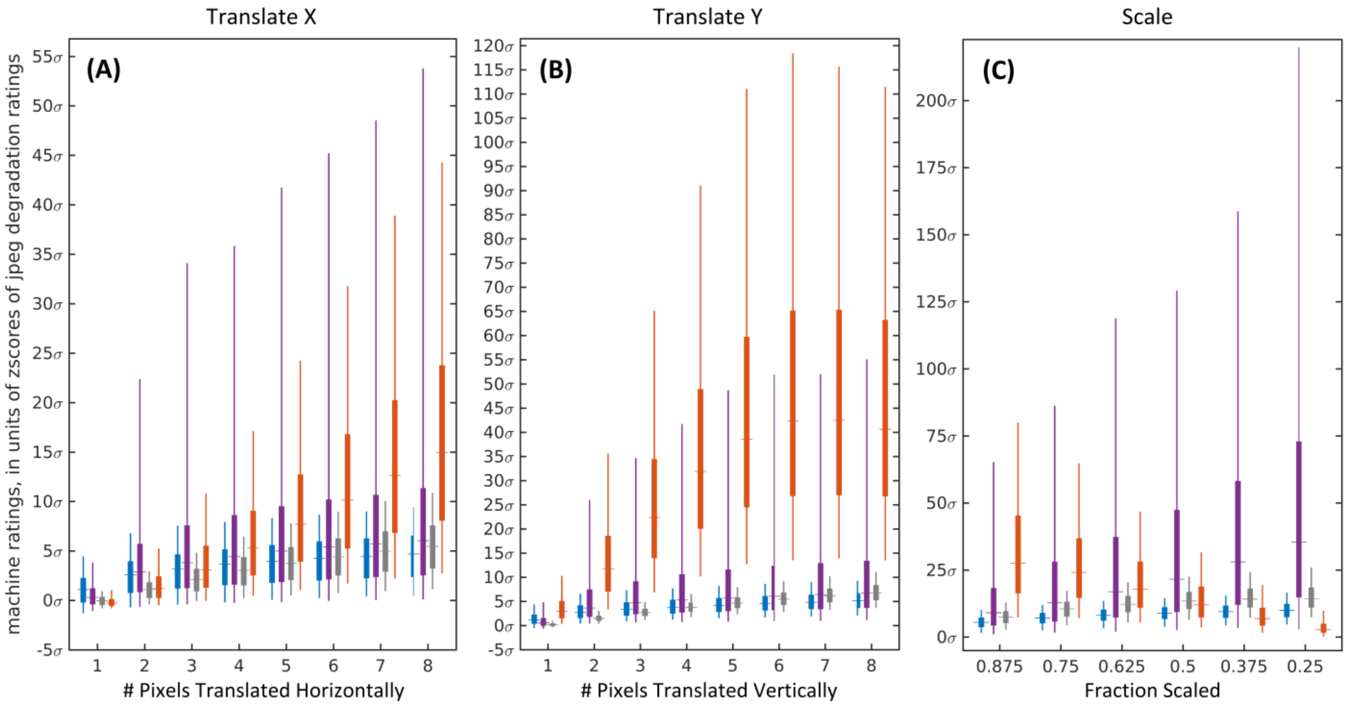

Supplemental Fig S 15. Invariance of various IQA models to **(A)** horizontal (rightward) translation, **(B)** vertical (downward) translation, and **(C)** (reduced) scale. Half of the SceneIQ Online dataset. In each plot, images are changed more from the original as the x axis moves right. Each distance measure – Euclidean distance (blue), SSIM (purple), approach **I** with Gaussian σ = 2.0 pixels (gray), approach **II** trained on the other half of images (orange), is plotted on the y axis as z scores of the same measures distance between reference and JPEG-degraded images. The boxes span from the 25th to 75th percentiles, and the medians are marked by horizontal lines. Lines span from the 5th to 95th percentiles.

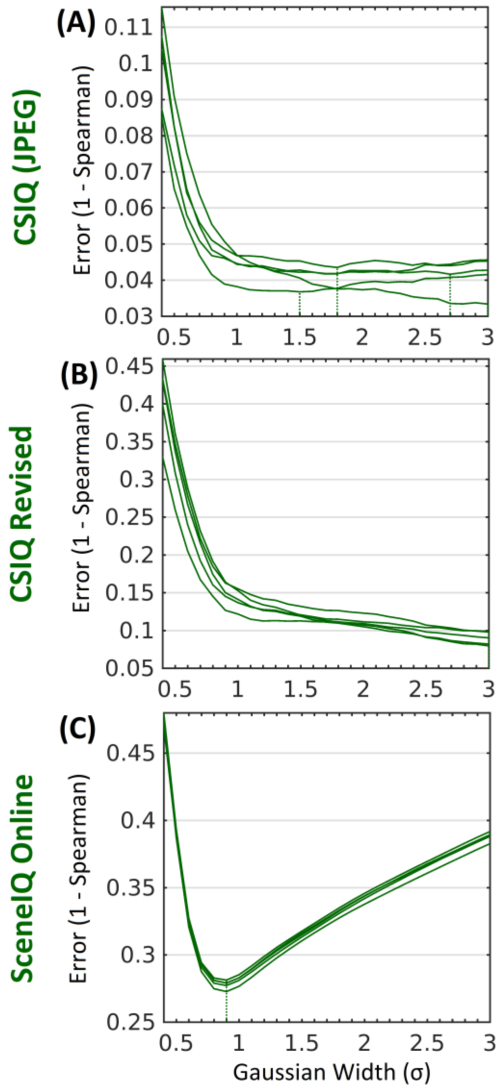

Supplemental Fig S 16. Approach **I** optimality with various Gaussian widths. A range of Gaussian widths (σ) were evaluated for each of five random folds of the **(A)** CSIQ (JPEG), **(B)** CSIQ Revised, and **(C)** SceneIQ Online datasets, using Spearman correlation. Dashed lines mark the global minima of each fold.

REFERENCES

Chandler, D. M., & Hemami, S. S. (2007). VSNR: A wavelet-based visual signal-to-noise ratio for natural images. *IEEE transactions on image processing, 16*, 2284-2298.

Cheng, G., Huang, J., Zhu, C., Liu, Z., & Cheng, L. (2010). Perceptual image quality assessment using a geometric structural distortion model. *Image Processing (ICIP), 2010 17th IEEE International Conference on*, (pp. 325-328).

Ferzli, R., & Karam, L. J. (2009). A no-reference objective image sharpness metric based on the notion of just noticeable blur (JNB). *IEEE transactions on image processing, 18*, 717-728.

Gao, X., Lu, W., Tao, D., & Li, X. (2009). Image quality assessment based on multiscale geometric analysis. *IEEE Transactions on Image Processing, 18*, 1409-1423.

Group, V. Q. (2000). *Final Report From the Video Quality Experts Group on the Validation of Objective Models of Video Quality Assessment.* Tech. rep., ITU. Retrieved from http://www.vqeg.org

Kim, D.-O., Han, H.-S., & Park, R.-H. (2010). Gradient information-based image quality metric. *IEEE Transactions on Consumer Electronics, 56*, 930-936.

Lagarias, J. C., Reeds, J. A., Wright, M. H., & Wright, P. E. (1998). Convergence properties of the Nelder--Mead simplex method in low dimensions. *SIAM Journal on optimization, 9*, 112-147.

Larson, E. C., & Chandler, D. M. (2010). Most apparent distortion: full-reference image quality assessment and the role of strategy. *Journal of Electronic Imaging, 19*, 11006-11006.

Moorthy, A. K., & Bovik, A. C. (2010). A two-step framework for constructing blind image quality indices. *IEEE Signal processing letters, 17*, 513-516.

Sheikh, H. R., Bovik, A. C., & De Veciana, G. (2005). An information fidelity criterion for image quality assessment using natural scene statistics. *IEEE Transactions on image processing, 14*, 2117-2128.

Sheikh, H. R., Sabir, M. F., & Bovik, A. C. (2006). A statistical evaluation of recent full reference image quality assessment algorithms. *IEEE Transactions on image processing, 15*, 3440-3451.

Wang, Z., Bovik, A. C., Sheikh, H. R., & Simoncelli, E. P. (2004). Image quality assessment: from error visibility to structural similarity. *IEEE transactions on image processing, 13*, 600-612.

Wang, Z., Simoncelli, E. P., & Bovik, A. C. (2003). Multiscale structural similarity for image quality assessment. *Signals, Systems and Computers, 2004. Conference Record of the Thirty-Seventh Asilomar Conference on, 2*, pp. 1398-1402.
